# Self‐Assembled Aza‐Boron‐Dipyrromethene‐Based H_2_S Prodrug for Synergistic Ferroptosis‐Enabled Gas and Sonodynamic Tumor Therapies

**DOI:** 10.1002/advs.202309542

**Published:** 2024-06-13

**Authors:** Jiajia Zhao, Erbao Bian, Renwu Zhang, Tao Xu, Yang Nie, Linqi Wang, Gui Jin, Han Xie, Huijing Xiang, Yu Chen, Dejun Wu

**Affiliations:** ^1^ Department of Neurosurgery The Second Affiliated Hospital of Anhui Medical University Hefei 230601 P. R. China; ^2^ Department of Neurosurgery Changzheng Hospital Naval Medical University Shanghai 200003 P. R. China; ^3^ School of Life Sciences Shanghai University Shanghai 200444 P. R. China; ^4^ Oujiang Laboratory (Zhejiang Lab for Regenerative Medicine, Vision and Brain Health) Wenzhou Institute of Shanghai University Wenzhou 325088 P. R. China; ^5^ Shanghai Institute of Materdicine Shanghai 200051 P. R. China

**Keywords:** ferroptosis, gas therapy, glioma treatment, nanomedicine, sonodynamic therapy

## Abstract

Glioblastoma multiforme (GBM) is the most aggressive and lethal subtype of gliomas of the central nervous system. The efficacy of sonodynamic therapy (SDT) against GBM is significantly reduced by the expression of apoptosis‐inhibitory proteins in GBM cells. In this study, an intelligent nanoplatform (denoted as Aza‐BD@PC NPs) based on the aza‐boron‐dipyrromethene dye and phenyl chlorothionocarbonate‐modified DSPE‐PEG molecules is developed for synergistic ferroptosis‐enabled gas therapy (GT) and SDT of GBM. Once internalized by GBM cells, Aza‐BD@PC NPs showed effective cysteine (Cys) consumption and Cys‐triggered hydrogen sulfide (H_2_S) release for ferroptosis‐enabled GT, thereby disrupting homeostasis in the intracellular environment, affecting GBM cell metabolism, and inhibiting GBM cell proliferation. Additionally, the released Aza‐BD generated abundant singlet oxygen (^1^O_2_) under ultrasound irradiation for favorable SDT. In vivo and in vitro evaluations demonstrated that the combined functions of Cys consumption, H_2_S production, and ^1^O_2_ production induced significant death of GBM cells and markedly inhibited tumor growth, with an impressive inhibition rate of up to 97.5%. Collectively, this study constructed a cascade nanoreactor with satisfactory Cys depletion performance, excellent H_2_S release capability, and prominent reactive oxygen species production ability under ultrasound irradiation for the synergistic ferroptosis‐enabled GT and SDT of gliomas.

## Graphical abstract

An intelligent nanoplatform (denoted as Aza‐BD@PC NPs) based on the aza‐boron‐dipyrromethene dye and phenyl chlorothionocarbonate‐modified DSPE‐PEG molecules is engineered for synergistic ferroptosis‐enabled gas therapy and sonodynamic therapy of gliomas. This study offers an alternative avenue to design innovative nanoplatforms to induce synergistic apoptosis and ferroptosis for effective gliomas treatment.

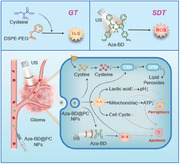

## 1 Introduction

Glioblastoma multiforme (GBM) is the most aggressive type of glioma of the central nervous system and is characterized by high morbidity, recurrence, and mortality rates.^[^ ^1^ ^]^ Despite advances in surgical resection and adjuvant therapies, such as combined radiotherapy and chemotherapy, GBM treatment remains unsatisfactory owing to the high recurrence rate and median survival of only 14 months.^[^ ^2^ ^]^ Sonodynamic therapy (SDT) is a promising treatment modality that utilizes ultrasound (US) to activate sonosensitizers for radical production, such as singlet oxygen (^1^O_2_), thereby inducing the apoptosis of glioma cells.^[^ ^3^ ^]^ Compared to therapies involving external stimuli,^[^ ^4^ ^]^ including photodynamic therapy (PDT)^[^ ^5^ ^]^ and magnetic field‐mediated cancer therapy,^[^ ^6^ ^]^ SDT offers various advantages, including precise control of the treatment area, non‐invasiveness, minimal side effects, favorable biocompatibility, and high penetration depth.^[^ ^7^ ^]^ However, the high expression of apoptosis‐suppressor proteins in GBM cells directly limits the efficacy of SDT against gliomas by impeding apoptosis‐related pathways.^[^ ^8^ ^]^ Therefore, it is imperative to identify alternative non‐apoptotic mechanisms of tumor cell death to improve the therapeutic efficacy of SDT.

Gas therapy (GT) is an innovative and promising cancer treatment strategy involving the use of various gas molecules to modulate cellular activity and affect disease progression.^[^ ^9^ ^]^ These gases include carbon monoxide (CO), nitric oxide (NO), hydrogen (H_2_), and hydrogen sulfide (H_2_S). H_2_S fights cancer by affecting glycolysis‐based energy production pathways via the “Warburg effect.”^[^ ^9^, ^10^ ^]^ At higher concentrations, H_2_S can affect mitochondrial function by disrupting the mitochondrial electron transport chain through its effect on cytochrome c oxidase, leading to pro‐oxidative stress. In addition, H_2_S induces cellular DNA damage.^[^ ^9^, ^11^ ^]^ Recent studies have explored the combination of SDT with GT by mechanically combining different components of a gas‐releasing prodrug and a sonosensitizer.^[^ ^12^ ^]^ This approach produces both gases and reactive oxygen species (ROS) under US irradiation. However, this combination lacks programmability, which limits its ability to adequately optimize both therapeutic outcomes.^[^ ^13^ ^]^ The cascade‐amplified killing effect on cancer cells cannot be achieved without the programmed generation of H_2_S and ROS.^[^ ^14^ ^]^ Thus, there is an urgent need to develop a gas‐releasing sonosensitizer that can be programmed to produce H_2_S and ROS while ensuring high biosafety.

Ferroptosis, a form of cell death observed in cancer cells, is distinct from apoptosis and is caused by excess lipid peroxidation (LPO) through an iron‐dependent regulatory mechanism.^[^ ^15^ ^]^ Nearest, ferroptosis has garnered significant attention as a promising area of research for the treatment of various cancers including kidney, liver, pancreatic, and ovarian cancers.^[^ ^16^ ^]^ Glutathione peroxidase 4 (GPX4), a major selenoprotein, is overexpressed in multiple cancers and plays a crucial role in inhibiting ferroptosis by protecting cells from LPO.^[^ ^17^ ^]^ There is growing evidence that the inhibition of GPX4 activity leads to LPO accumulation, induces ferroptosis, and suppresses tumor growth.^[^ ^18^ ^]^ In a recent study, the direct depletion of glutathione (GSH) reduced GPX4 activity, resulting in an increased cumulative LPO effect and enhanced ferroptosis.^[^ ^7^, ^19^ ^]^ GSH is a tripeptide consisting of cysteine (Cys), glutamate, and glycine; Cys is the key rate‐limiting precursor in its synthesis.^[^ ^20^ ^]^ Thus, Cys depletion directly suppresses GSH biosynthesis,^[^ ^21^ ^]^ resulting in more severe ferroptosis than GSH depletion.^[^ ^19^, ^22^ ^]^

**Figure 1:**
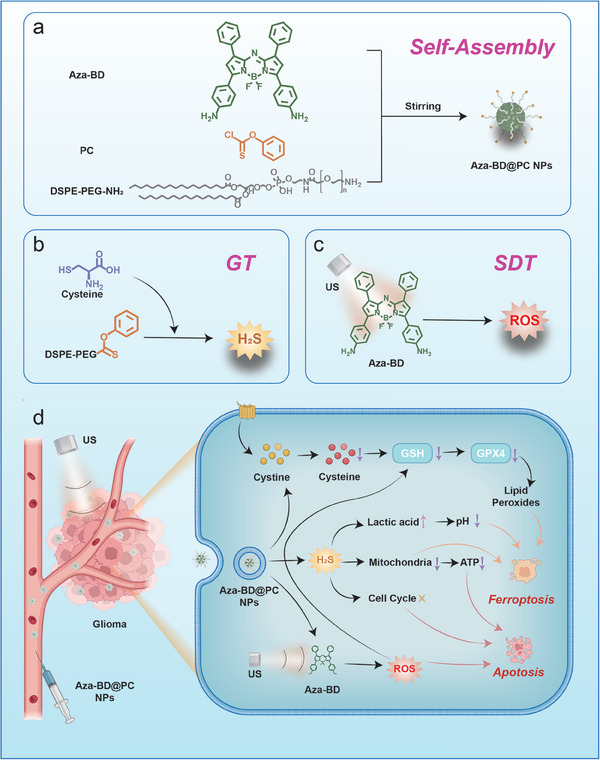
Fabrication of Aza‐BD@PC NPs and their utilization in synergistic GT and SDT. a) Fabrication of Aza‐BD@PC NPs. b) H_2_S produced by Aza‐BD@PC NPs for GT in the presence of Cys. c) ^1^O_2_ production of Aza‐BD@PC NPs plus US stimulation for SDT. d) Synergistic GT and SDT effect caused by Aza‐BD@PC NPs under US irradiation for enhanced therapeutic efficacy.

In the present study, we engineered a specific Cys‐triggered intelligent multifunctional nanoplatform (Aza‐BD@PC NPs) to achieve Cys depletion, acidosis induction, and intratumoral metabolic symbiosis disruption for synergistic GT and ferroptosis‐augmented SDT in GBM. The biocompatible polymer PC‐modified DSPE‐PEG is self‐assembled with Aza‐BD to form the nanoplatform Aza‐BD@PC NPs. Once internalized by GBM cells, Aza‐BD@PC NPs selectively and effectively deplete intracellular Cys by mild cleavage of S─N bonds. This process hinders GSH synthesis and produces H_2_S, thereby disrupting the intracellular redox balance, affecting glycolytic pathways, and subsequently promoting ferroptotic cell death. In addition, H_2_S causes DNA damage and inhibits the proliferation of GBM cells. Furthermore, the released sonosensitizer Aza‐BD produces abundant ^1^O_2_ under US irradiation, thereby promoting effective SDT. In vivo experiments using GL261 tumor‐bearing mice verified the collaborative antitumor effect of the combination of GT and ferroptosis‐augmented SDT (**Figure** **1**). Therefore, this study not only presents a prospective strategy for the development of a cascade nanoreactor to induce synergistic apoptosis and ferroptosis for treating GBM but also provides an alternative avenue to overcome the barriers that hinder the effectiveness of GBM treatment.

## 2 Results and Discussion

### 2.1 Synthesis and Characterization of Aza‐BD@PC NPs

**Figure 2:**
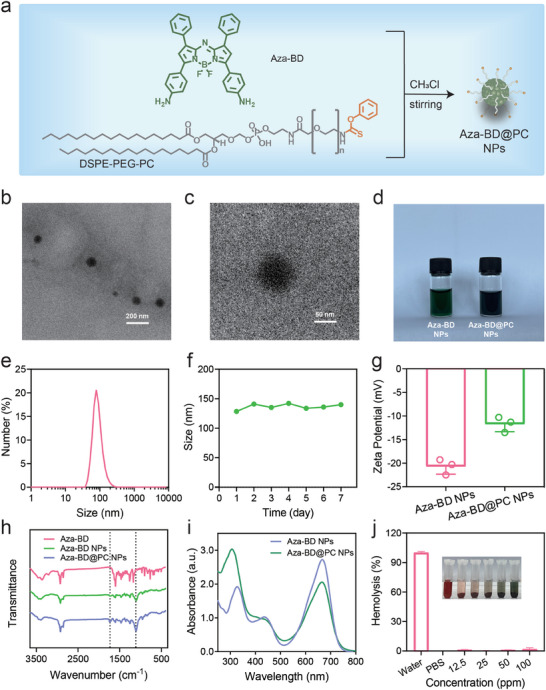
Fabrication and characterization of Aza‐BD@PC NPs. a) The synthetic route of Aza‐BD@PC NPs. b,c) TEM images of Aza‐BD@PC NPs at different magnifications. d) Digital image of Aza‐BD NPs and Aza‐BD@PC NPs in PBS. e) Size distribution of Aza‐BD@PC NPs as measured using DLS. f) Size distribution of Aza‐BD@PC NPs in PBS over 7 days. g) Zeta potential of Aza‐BD NPs and Aza‐BD@PC NPs (n = 3). h) FTIR spectra of Aza‐BD, Aza‐BD NPs, and Aza‐BD@PC NPs. i) UV/vis spectra of Aza‐BD NPs and Aza‐BD@PC NPs aqueous solutions. j) Hemolysis rates of Aza‐BD@PC NPs at various concentrations (n = 3). Data are represented as mean ± SD.

The detailed synthetic route and related characterization for Aza‐BD and PC‐modified DSPE‐PEG‐NH_2_ are provided in Scheme S1 and Figures S1 and S2 (Supporting Information). To improve the water solubility and biocompatibility of Aza‐BD, Aza‐BD@PC NPs were fabricated by self‐assembling an amphiphilic prodrug of H_2_S gas (DSPE‐PEG‐PC) and sonosensitizer molecules (Aza‐BD) via nanoprecipitation (**Figure** **2**a). The morphology and size of the Aza‐BD@PC NPs were examined using transmission electron microscopy (TEM). TEM images showed that the Aza‐BD@PC NPs were monodisperse and spherical, with an average size of ≈80 nm (Figure 2b,c). Aza‐BD and Aza‐BD@PC NPs dispersed in phosphate‐buffered saline (PBS) were observed as blue‐green, transparent colloidal dispersions (Figure 2d). Dynamic light scattering (DLS) measurements revealed that the hydrodynamic diameter of Aza‐BD@PC was 130 nm (Figure 2e). In addition, the size of the Aza‐BD@PC NPs remained stable during the 7‐day observation period and did not show significant changes, indicating that the NPs were highly stable under physiological conditions (Figure 2f). The zeta potentials of Aza‐BD and Aza‐BD@PC NPs were examined to assess their surface charges. After the conjugation of the PC molecule with DSPE‐PEG‐NH_2_, the introduction of the PC moiety caused an increase in the surface potential from −20.7 to −11.7 mV (Figure 2g). The change in the zeta potential verified the successful construction of Aza‐BD@PC NPs. Furthermore, the Fourier‐transform infrared (FTIR) spectrum of the Aza‐BD@PC NPs exhibited typical peaks at 1114 and 1733 cm^−1^, corresponding to the vibrational peaks of the C─H and C─O bonds of Aza‐BD, respectively (Figure 2h). These peaks further confirmed the successful establishment of Aza‐BD@PC NPs. The UV–vis–NIR spectrum of Aza‐BD@PC NPs exhibited high absorbance at 670 nm (Figure 2i). In addition, the hemolytic behavior of the Aza‐BD@PC NPs was assessed to validate their biocompatibility. Negligible hemolysis (hemolysis rate < 5%) was observed at a concentration of 100 ppm, confirming the high hemocompatibility of Aza‐BD@PC NPs (Figure 2j).

### 2.2 Controllable Release of ^1^O_2_ and H_2_S of Aza‐BD@PC NPs Under US irradiation

**Figure 3:**
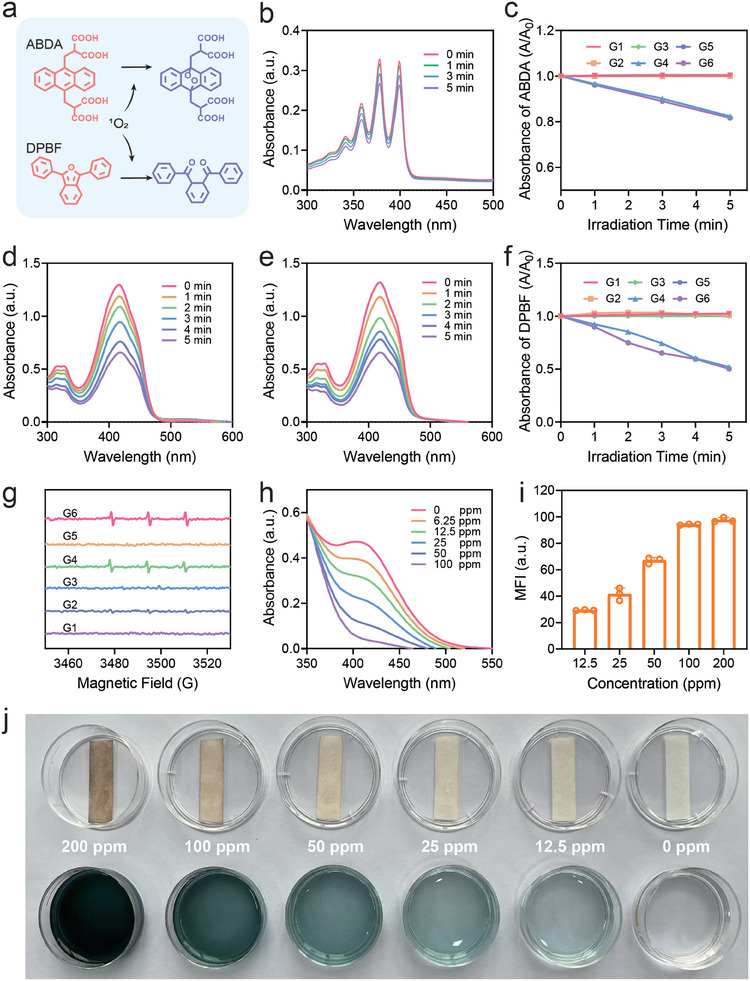
^1^O_2_ and H_2_S production of Aza‐BD@PC NPs plus US actuation. a) Schematic diagram of ^1^O_2_ detection using ^1^O_2_ indicators, such as ABDA and DPBF. b) UV–vis spectra of ABDA aqueous solution containing Aza‐BD@PC NPs under US irradiation for different durations. c) Relative variations in the absorption intensities of ABDA at 400 nm in various treatment groups (n = 3). d,e) UV–vis spectra of DPBF solution containing (d) Aza‐BD NPs and (e) Aza‐BD@PC NPs under US irradiation for different durations. f) Relative variations in the absorption intensities of DPBF at 415 nm in different treated groups (n = 3). g) ESR spectra in different treatment groups using TEMP as a ^1^O_2_ trapping agent. h) UV–vis absorption spectra of DTNB aqueous solutions after addition of Aza‐BD@PC NPs in the presence Cys at diverse concentrations. i) Mean fluorescence intensities of Aza‐BD@PC NPs in the presence of diverse doses of Cys using WSP‐1 as a H_2_S probe (n = 3). j) Detection of H_2_S release generated by Aza‐BD@PC NPs in the presence of different concentrations of Cys using lead acetate test paper. G1: control, G2: US, G3: Aza‐BD NPs, G4: Aza‐BD NPs + US, G5: Aza‐BD@PC NPs, G6: Aza‐BD@PC NPs + US. Data are represented as mean ± SD.

The ability of Aza‐BD@PC NPs to produce ^1^O_2_ under US stimulation was assessed using 9,10‐anthracenediyl‐bis(methylene) dimalonic acid (ABDA) as a probe (**Figure** **3**a‐c; Figure S3, Supporting Information). The absorption intensity of ABDA at 400 nm decreased after oxidation with ^1^O_2_. In the Aza‐BD NPs + US and Aza‐BD@PC NPs + US groups, the ABDA absorbance decreased with prolonged US irradiation time, resulting in an I/I_0_ value of 0.2, confirming the effective generation of ^1^O_2_ by Aza‐BD under US irradiation. However, the decrease in the ABDA absorbance at 400 nm was negligible in the Aza‐BD, Aza‐BD@PC, and US groups (Figure S3, Supporting Information). Subsequently, 1,3‐diphenylbenzofuran (DPBF) was used as an ^1^O_2_ indicator to further assess the capability of Aza‐BD@PC NPs to produce ^1^O_2_ under US irradiation, and the corresponding results were consistent with those obtained using ABDA (Figure 3d‐f; Figure S4, Supporting Information). Moreover, the ^1^O_2_ quantum yield of Aza‐BD NPs and Aza‐BD@PC NPs under US irradiation was determined to be 90% and 91%, respectively, using methylene blue (MB) as a control (Figure S5, Supporting Information). Additionally, ^1^O_2_ production by Aza‐BD@PC NPs under US actuation was verified by electron spin resonance (ESR) using 2,2,6,6‐tetramethylpiperidine (TEMP) as the trapping reagent (Figure 3g). A distinct three‐line ESR peak of the ^1^O_2_/TEMP adduct with a relative intensity of 1:1:1 was detected in the Aza‐BD NPs + US and Aza‐BD@PC NPs + US groups, indicating that both Aza‐BD NPs and Aza‐BD@PC NPs could efficiently generate ^1^O_2_ under US irradiation.

To detect H_2_S release from Aza‐BD@PC NPs in response to Cys, Ellman's reagent and Washington State Probe‐1 (WSP‐1) were used as detection probes for Cys and H_2_S, respectively (Figure 3h,i). The results demonstrated that Aza‐BD@PC NPs consumed Cys and produced H_2_S in a concentration‐dependent manner, confirming efficient H_2_S production by Aza‐BD@PC NPs in the presence of Cys. Additionally, the ability of the Aza‐BD@PC NPs to produce H_2_S gas was examined using a lead acetate test paper as an indicator that can capture H_2_S to generate black lead sulfide (PbS). After the addition of Cys, the darker color of the solution qualitatively indicated a higher H_2_S concentration (Figure 3j). These results illustrate that Aza‐BD@PC NPs effectively released H_2_S in a dose‐dependent manner in the presence of Cys.

### 2.3 In Vitro Therapeutic Efficacy of Aza‐BD@PC NPs Under US Irradiation

**Figure 4:**
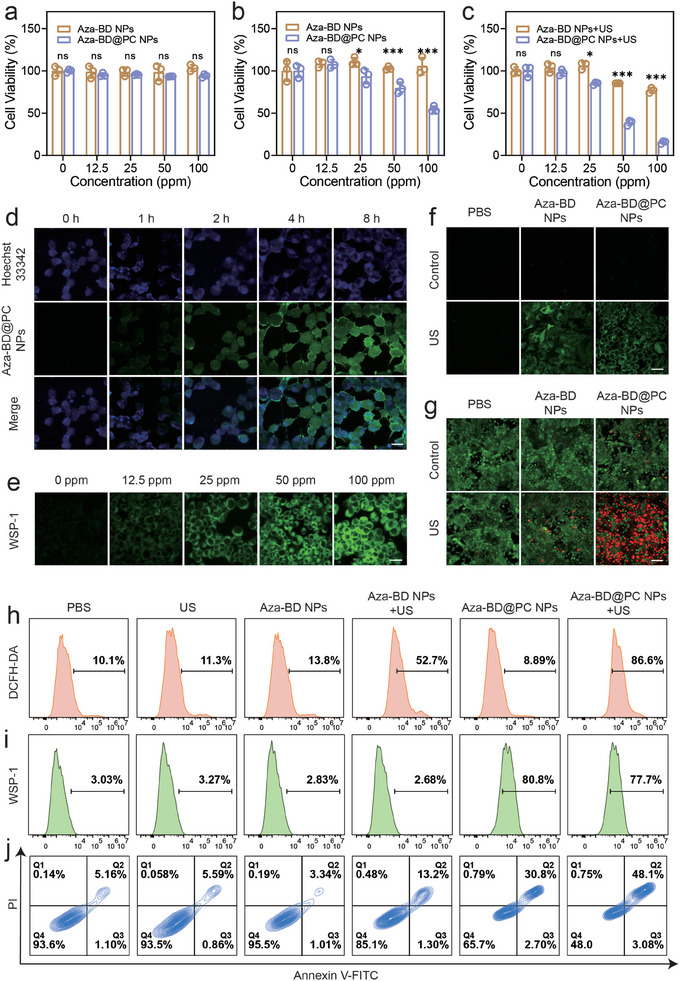
Treatment efficacy of Aza‐BD@PC NPs under US exposure in vitro. a,b) Cell viabilities of (a) 3T3 cells and (b) GL261 cells treated with different doses of Aza‐BD NPs and Aza‐BD@PC NPs (n = 5). c) Survival rates of GL261 cells after treatment with different doses of Aza‐BD@PC NPs under both US and non‐US conditions (n = 5). d) Confocal images of GL261 cells after incubated with FITC‐labeled Aza‐BD@PC‐ NPs for various incubation durations. Scale bar: 30 µm. e) Confocal images of GL261 cells stained with WSP‐1 after incubated with different concentrations of Aza‐BD@PC NPs. Scale bar: 50 µm. f,g) CLSM images of GL261 cells stained with (f) DCFH‐DA and (g) calcein‐AM/PI after various treatments (scale bars: 50 µm for f and 100 µm for g, respectively). h–j) Flow cytometry analysis of GL261 cells stained with (h) DCFH‐DA, (i) WSP‐1, and (j) Annexin V‐FITC/PI in diverse treated groups. Data are represented as mean ± SD and analyzed by one‐way ANOVA. ns: no statistical difference, ^*^ *p* < 0.05, ^**^ *p* < 0.01, and ^***^ *p* < 0.001.

Prior to performing in vitro evaluation, it was essential to validate whether Aza‐BD@PC NPs could be effectively internalized by GL261 cells. Fluorescein 5‐isothiocyanate (FITC)‐labeled Aza‐BD@PC NPs were used to evaluate the cellular endocytosis of Aza‐BD@PC NPs at different incubation time points. Confocal laser scanning microscopy (CLSM) images showed that the green fluorescence signal of Aza‐BD@PC‐FITC NPs in GL261 cells increased with prolonged incubation, demonstrating that the cellular uptake of Aza‐BD@PC NPs exhibited an incubation‐duration‐dependent pattern (**Figure** **4**d). Flow cytometry analysis was conducted to quantitatively investigate the cellular endocytosis of Aza‐BD@PC NPs. The green fluorescence levels in GL261 cells were 14.0% and 51.7% after incubation for 1 and 4 h, respectively, which was consistent with the CLSM observations (Figure S6, Supporting Information).

Subsequently, we performed the cell counting kit‐8 (CCK‐8) assay to evaluate the toxicity of Aza‐BD and Aza‐BD@PC NPs toward glioma cells. Notably, no significant cell death was observed in mouse embryonic fibroblasts (3T3) and GL261 cells after treatment with Aza‐BD NPs. However, treatment with Aza‐BD@PC NPs resulted in significantly lower survival of GL261 cells than that of 3T3 cells (Figure 4a,b). This difference is mainly attributed to the high redox levels in GBM cells, which constantly trigger the release of H_2_S, thereby leading to more severe damage to GL261 cells. The CCK‐8 assay results for Aza‐BD and Aza‐BD@PC NPs confirmed the killing effect of GT on GL261 cells. Furthermore, we performed a standard CCK‐8 assay on GL261 cells after combined US irradiation to investigate the effects of SDT on cell death. Importantly, the relative viabilities of GL261 cells in the Aza‐BD NPs and Aza‐BD@PC NPs groups were ≈100% and 54.61%, respectively, at a concentration of 100 ppm. After US irradiation, the cellular survival rate decreased to ≈77.04% and 15.58% in the Aza‐BD NPs + US and Aza‐BD@PC NPs + US groups, respectively (Figure 4c). Additionally, the in vitro cytotoxicity of Aza‐BD@PC NPs on 4T1 breast cancer cells under US irradiation was investigated. Compared with the control group, the cell viability decreased to 54.26% after incubation with 100 ppm of Aza‐BD@PC NPs and US irradiation for 5 min (Figure S7, Supporting Information). These observations suggested that the combination of H_2_S‐mediated GT and ^1^O_2_‐mediated SDT induced greater cytotoxic effects than either treatment alone.

Intracellular H_2_S generation was assessed by CLSM using WSP‐1 as the fluorescent probe. CLSM images revealed that H_2_S production in GL261 cells was dose‐dependent (Figure 4e). In addition, GL261 cells from various treatment groups were subjected to flow cytometry. Strong green fluorescence signals with intensities of 2.83% and 80.80% were detected in the Aza‐BD NPs + US and Aza‐BD@PC NPs + US groups, respectively, demonstrating the high capability of Aza‐BD@PC NPs to produce H_2_S in GL261 cells (Figure 4i). We then used the 2’,7’‐dichlorodihydrofluorescein diacetate (DCFH‐DA) probe to determine the ROS levels in the differently treated cells. The results of CLSM observation and flow cytometry analysis showed that ROS production was detected in the Aza‐BD NPs + US and Aza‐BD@PC NPs + US groups, indicating that Aza‐BD and Aza‐BD@PC NPs produced abundant ROS that killed GL261 cells under US irradiation (Figure 4f,h).

To confirm the cytotoxicity of Aza‐BD@PC NPs triggered by US, we conducted a calcein acetoxymethyl ester/propidium iodide (calcein‐AM/PI) cell viability assay to observe live and dead GL261 cells in various groups using CLSM (Figure 4g). Strong fluorescent calcein‐AM signals were detected in the control, US, and Aza‐BD NP groups. In contrast, the GL261 cells in the Aza‐BD@PC and Aza‐BD NPs + US groups displayed both green and red fluorescent signals. Importantly, a distinct red fluorescence signal and a weak green fluorescence signal were detected in the Aza‐BD@PC NPs + US group, confirming that synergistic GT and SDT exerted a significant cytotoxic effect on GL261 cells. Furthermore, an annexin V‐FITC/PI staining assay was conducted to analyze the Aza‐BD@PC NP‐induced apoptosis under US irradiation using flow cytometry. Cells treated with 50 ppm Aza‐BD@PC NPs showed limited antitumor effects, resulting in an apoptosis rate of 33.5% (Figure 4j). In addition, an apoptosis rate of 14.5% was observed in GL261 cells treated with the same dose of Aza‐BD NPs under US irradiation. Notably, 50 ppm Aza‐BD@PC NPs had an ideal therapeutic effect on GL261 cells with an apoptosis ratio of 51.2% under US irradiation, suggesting that the combination of GT and SDT induced significant apoptosis by the simultaneous generation of H_2_S and ^1^O_2_ under US irradiation.

### 2.4 Related Mechanism Exploration In Vitro

**Figure 5:**
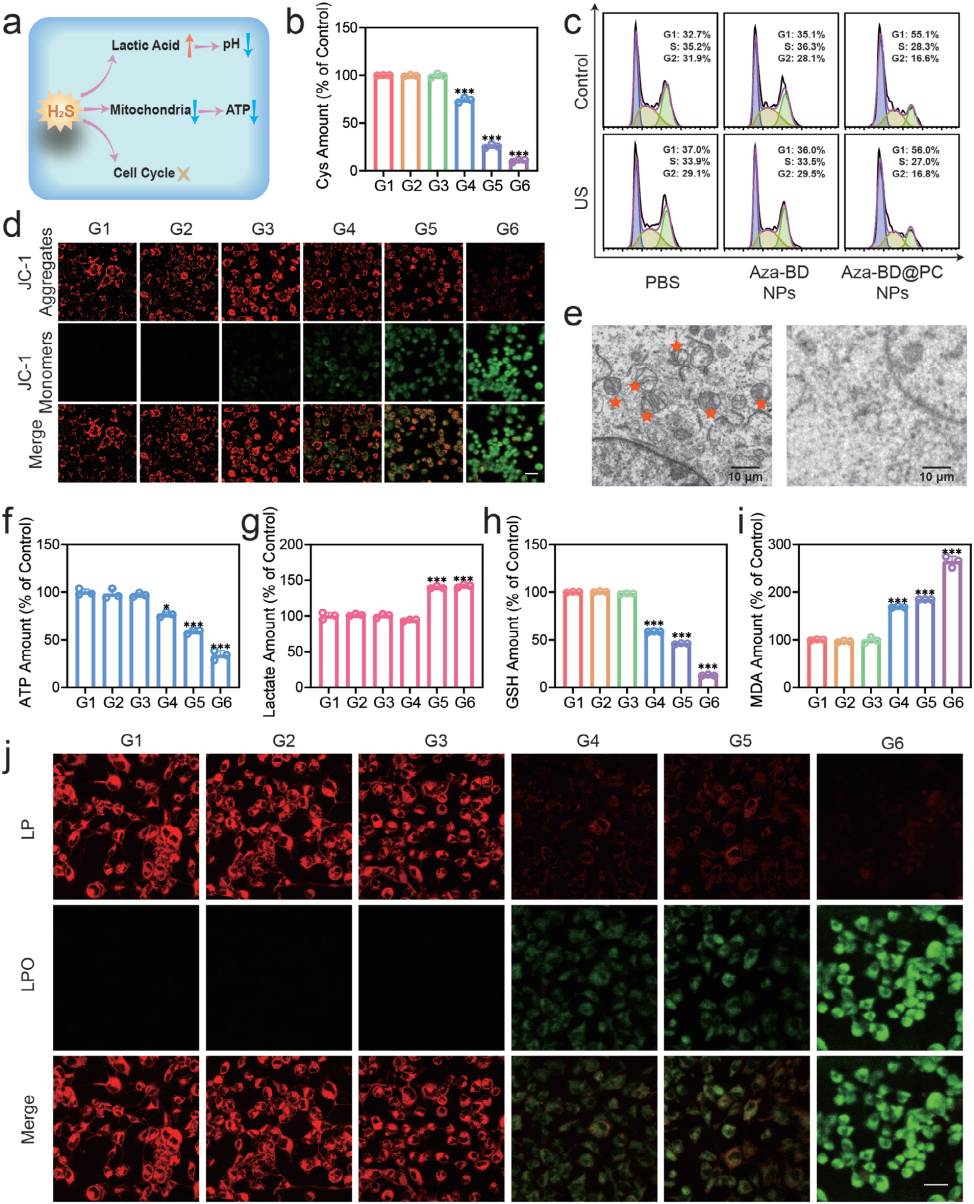
Intracellular H_2_S generation and ferroptosis induction of Aza‐BD@PC NPs under US irradiation. a) Schematic diagram of the mechanism by which H_2_S affects cell function. b) The relative concentration of intracellular Cys after diverse treatments (n = 5). c) Flow cytometry cell cycle analysis of GL261 cells labeled with PI in various treatment groups. d) CLSM images of JC‐1 aggregates (red) and JC‐1 monomers (green) in GL261 cells after diverse treatments using the JC‐1 probe (scale bar: 50 µm). e) Bio‐TEM images of GL261 cells in the control and Aza‐BD@PC NPs + US groups. Stars indicate the mitochondria within the cell. Scale bar: 10 µm. f) ATP, g) lactic acid, h) GSH, and i) MDA levels in GL261 cells after various treatment (n = 3). j) CLSM images of LP (red) and LPO (green) in GL261 cells after various treatment using the LPO probe. Scale bar: 50 µm. G1: control, G2: US, G3: Aza‐BD NPs G4: Aza‐BD NPs + US, G5: Aza‐BD@PC NPs, G6: Aza‐BD@PC NPs + US. Data are represented as mean ± SD and analyzed by one‐way ANOVA. ns: no statistical difference, ^*^ *p* < 0.05, ^**^ *p* < 0.01, and ^***^ *p* < 0.001.

The mechanism of cell death induced by Aza‐BD@PC NPs in GL261 cells was investigated. The depletion of intracellular Cys by Aza‐BD@PC NPs under US irradiation was verified using a Cys kit. A significant decrease in Cys content was detected in the Aza‐BD@PC NPs and Aza‐BD@PC NPs + US groups compared with that in the control group, indicating the excellent ability of Aza‐BD@PC NPs to deplete Cys (**Figure** **5**b). Previous studies have shown that H_2_S can inhibit tumor cell division by affecting the cell cycle and cell growth (Figure 5a). Therefore, the Cell Cycle and Apoptosis Analysis kit was used to evaluate the cell cycle of the tumor cells and verify the effect of Aza‐BD@PC NPs on cell proliferation. The G1 phase ratios in the Aza‐BD@PC NPs and Aza‐BD@PC NPs + US groups were 55.1% and 56.0%, respectively, which were higher than those in the other treatment groups. The results indicated that Aza‐BD@PC NPs efficiently generated H_2_S through the reaction between Aza‐BD@PC NPs and intracellular Cys, thereby affecting DNA replication and inhibiting the transition of cells from the G1 to the G2 phase (Figure 5c).

It has been demonstrated that H_2_S‐mediated inhibition of mitochondrial respiration and ATP production can lead to cell necrosis and apoptosis (Figure 5a).^[^ ^9c^ ^]^ To verify the induction of apoptosis, the mitochondrial membrane potential was assessed using a JC‐1 kit. The Aza‐BD@PC and Aza‐BD@PC NPs + US groups exhibited stronger green fluorescence, indicating impaired mitochondrial respiration caused by H_2_S and ROS production (Figure 5d). Bio‐TEM observations of GL261 cells in the Aza‐BD@PC NPs + US group further revealed that ROS and H_2_S produced by Aza‐BD@PC NPs under US irradiation detrimentally affected the mitochondria, thereby leading to tumor cell death (Figure 5e).

Moreover, the ATP assay demonstrated that the combined effect of excess H_2_S and ROS inhibited ATP generation, thereby inducing apoptosis (Figure 5f). Additionally, according to previous studies, H_2_S promotes glucose uptake and depletion to generate lactic acid (Figure 5a). Therefore, a lactate detection kit was used to measure the intracellular lactic acid content to verify the effect of Aza‐BD@PC NPs on cell metabolism under US irradiation. Higher lactate levels were detected in the Aza‐BD@PC NPs and Aza‐BD@PC NPs + US groups, indicating that Aza‐BD@PC NPs affect tumor lactate metabolism by producing H_2_S in the presence of intracellular Cys (Figure 5g). In addition, a BCECF‐AM assay was performed, and the results indicated that excess H_2_S in GL261 cells produced by Aza‐BD@PC NPs triggered a cascade of reactions, thereby acidifying tumor cells and affecting cellular lactate metabolism (Figure S8, Supporting Information). Therefore, Aza‐BD@PC NPs produced abundant H_2_S, inhibited mitochondrial respiration and ATP synthesis, and affected glycolysis and metabolic symbiosis, thereby inhibiting GL261 cell proliferation.

Considering that Aza‐BD@PC NPs can effectively consume intracellular Cys and that Cys depletion hinders GSH synthesis, we conducted a GSH assay to monitor intracellular GSH levels in different groups. The results showed that Cys depletion or ROS generation moderately reduced the GSH levels in GL261 cells, and the combined effect of both led to a more substantial reduction in GSH levels (Figure 5h). GSH depletion or ROS overproduction results in LPO, which is a typical feature of ferroptosis. We then measured the intracellular LPO levels by performing a malondialdehyde (MDA) assay to confirm the induction of ferroptosis by Aza‐BD@PC NPs under US irradiation. The results revealed that Cys consumption or ROS generation modestly reduced the antioxidant capacity of cells, leading to a detectable increase in LPO production, whereas a significant increase in LPO levels was detected via the combined effect of Cys depletion and ROS production (Figure 5i). We also used BODIPY581/591‐C11 to measure the LPO levels in GL261 cells. CLSM images revealed that GL261 cells in the Aza‐BD NPs + US and Aza‐BD@PC NPs groups displayed weaker green fluorescence than the other treatment groups. Aza‐BD NPs can produce ROS upon US irradiation to increase intracellular oxidative stress level (Figure 5j). In addition, PC‐modified DSPE‐PEG‐NH_2_ can increase intracellular oxidative stress level by decreasing intracellular Cys level. However, we observed significantly stronger green fluorescence in the Aza‐BD@PC NPs + US group, indicating that Aza‐BD@PC NPs can produce ROS and deplete intracellular Cys under US irradiation, thereby significantly increasing the level of oxidative stress. Western blot analysis was then carried out to investigate the expression level of GPX4 after various treatments (Figure S9, Supporting Information). The GPX4 expression level was markedly decreased in the Aza‐BD@PC NPs + US group compared with that in the other treatment groups, indicating enhanced ferroptosis induction triggered by Aza‐BD@PC NPs under US stimulation.

### 2.5 Mechanisms of Synergistic GT and CDT Mediated by Aza‐BD@PC NPs Under US Irradiation

**Figure 6:**
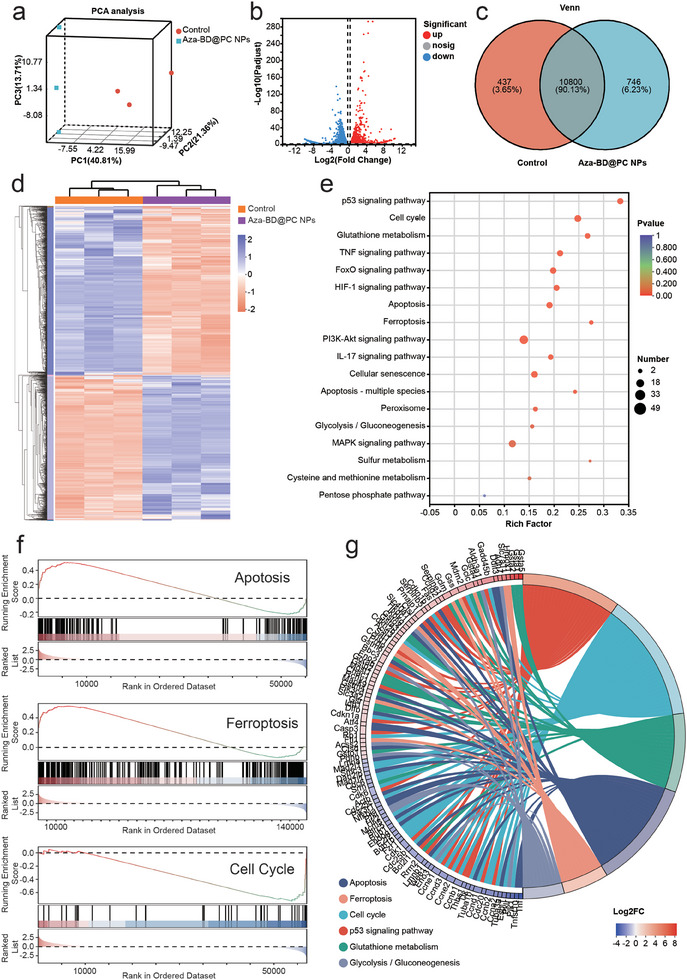
RNA‐seq analysis of the potential therapeutic mechanism after treated with Aza‐BD@PC NPs under US irradiation. a) Plot of PCA, b) Volcano plot for the DEGs in the control and Aza‐BD@PC NPs + US‐treated groups, FC ≥ 2.0 (or ‐2.0), *p* < 0.05. c) Venn plot for the number of DEGs in the control and Aza‐BD@PC NPs + US group. d) Heat map of the DEGs in the control and Aza‐BD@PC NPs + US groups. e) Bubble chart showing the DEGs enriched in the KEGG. f) GSEA of DEGs in the apoptosis, ferroptosis and cell cycle signaling pathways after treatment with Aza‐BD@PC NPs under US irradiation. g) KEGG chord diagram depicting the up‐regulated and down‐regulated genes in multiple signaling pathways.

To elucidate the latent anti‐glioma effect of Aza‐BD@PC NPs under US stimulation, we conducted RNA sequencing (RNA‐Seq) to analyze mRNA expression profiles in the control and Aza‐BD@PC NPs + US groups. Principal component analysis revealed significant differences in mRNA expression between the control and Aza‐BD@PC NPs + US groups (**Figure** **6**a). Compared with the control group, ≈7133 genes were dysregulated in GL261 cells in the Aza‐BD@PC NPs + US group, comprising 3541 upregulated and 3592 downregulated genes. The differentially expressed genes (DEGs) were determined using the screening criteria (Figure 6b,c). Subsequently, a heat map was created for the DEGs in both the control and Aza‐BD@PC NPs + US groups, and a difference in gene expression between the two groups was observed (Figure 6d).

Gene ontology analysis revealed that DEGs in both the control and Aza‐BD@PC NPs + US groups were significantly associated with multiple cellular biological processes, including glutathione metabolic processes, response to ROS, mitotic cell cycle phase transition, and intrinsic apoptotic signaling pathways (Figure S10, Supporting Information). In addition, the Kyoto Encyclopedia of Genes and Genomes analysis demonstrated that various biological pathways, including apoptosis, ferroptosis, and TNF‐and p53 cellular signaling pathways, were significantly enriched after treatment with Aza‐BD@PC NPs and US stimulation (Figure 6e). In addition, Gene Set Enrichment analysis illustrated significant changes in the expression levels of genes related to apoptosis and ferroptosis in the Aza‐BD@PC NPs + US group (Figure 6f). Chord analysis indicated that 95 genes participated in the enriched pathways, including the p53 signaling pathway, glutathione metabolism, cell cycle, apoptosis, ferroptosis, and glycolysis/gluconeogenesis pathways (Figure 6g). Fas and caspase‐3 have been shown to participate in p53 signaling and apoptosis pathways. Interestingly, tumor‐related genes such as Fas and caspase‐3 were distinctly upregulated after treatment with Aza‐BD@PC NPs *plus* US stimulation.^[^ ^23^ ^]^ Furthermore, several genes associated with ferroptosis, including SLC7A11, Gclc, GSS, and Slc40a1, were significantly upregulated (Figure 6g).^[^ ^24^ ^]^ Therefore, Aza‐BD@PC NPs mediate Cys consumption, H_2_S generation, and ROS generation under US actuation, collectively facilitating apoptosis and ferroptosis, resulting in the death of GL261 cells.

### 2.6 In Vivo Biocompatibility Evaluation

Prior to the in vivo studies, we used 15 healthy Kunming mice to assess the potential toxicity of Aza‐BD@PCNPs. Liver function, renal function, and blood biochemical results confirmed that the Aza‐BD@PC NPs were highly biocompatible (Figures S11–S13, Supporting Information). Unlike traditional drug delivery system (DDS), it has been demonstrated that self‐assembled polymers that covalently tethered sonosensitizers are therefore more stable and can avoid premature release.^[^ ^25^ ^]^ Self‐assembled polymers can increase the solubility of the parent drug as well as enable passive targeting of solid tumors. The self‐assembled polymers with nanoscale characteristics can be delivered to cells without the aid of excipients, and have multiple advantages, such as high drug loading, large molecular weight, controllable drug ratio, enhanced stability, and desirable synergistic outcome. Cancer tissues are rich in acidity, reducing condition, and overexpressed receptors in tumor microenvironment, and a vulnerable immune system, all of which are specific stimuli that trigger drug release. These features, along with enhanced permeability and retention (EPR) effect, are considered to underpin targeted cancer therapy for Aza‐BD@PC NPs. Furthermore, we investigated the biodistribution of Aza‐BD@PC NPs in mice bearing GL261 tumors using the in vivo imaging system (IVIS). IVIS images showed that the fluorescence intensity of the Aza‐BD@PC NPs at the glioma site gradually increased over time, reaching a maximum intensity 12 h after intravenous injection (Figure S14, Supporting Information). Additionally, fluorescence imaging of the isolated organs revealed that Aza‐BD@PC NPs mainly accumulated in the glioma tissues and major organs 24 h after intravenous injection (Figure S15, Supporting Information). These findings indicate that Aza‐BD@PC NPs have favorable biodistribution characteristics and specific accumulation in tumor tissues, guaranteeing their function as efficient therapeutic agents for cancer treatment.

### 2.7 In Vivo Therapeutic Performance of Aza‐BD@PC NPs Under US Irradiation

**Figure 7:**
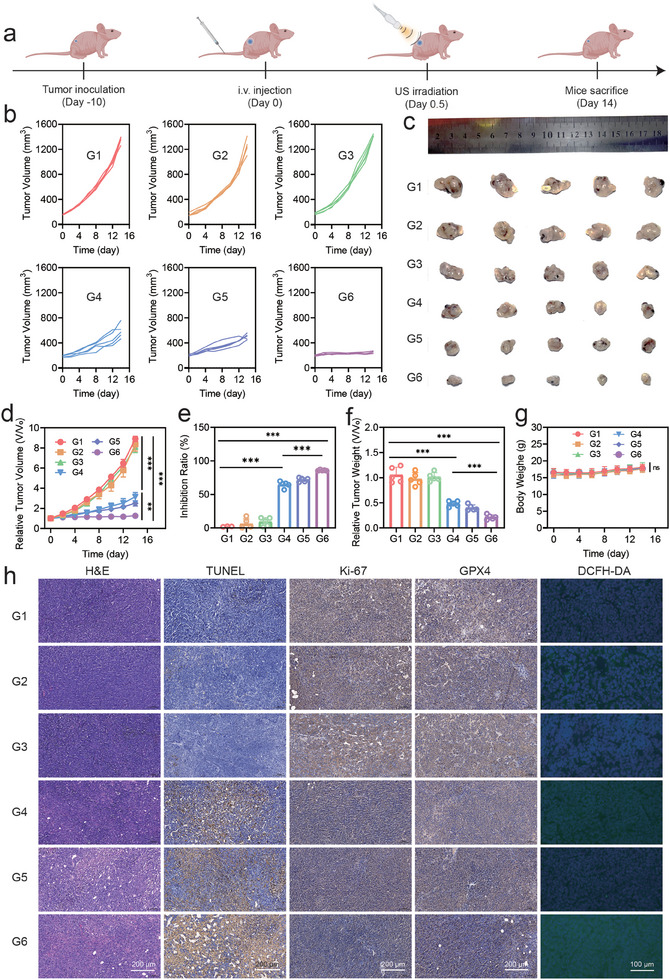
Treatment effectiveness of Aza‐BD@PC NPs *plus* US irradiation in vivo. a) The therapeutic schedule for GL261 tumor‐bearing mice. b) Growth curves of individual tumors, c) digital photographs of tumors, d) changes in tumor volumes, e) tumor‐growth inhibition value, f) weights of isolated tumors, and g) body weights of the mice from various groups (n = 5). h) H&E, TUNEL, Ki‐67, DCFH‐DA, and GPX4 staining images of glioma slices from diverse groups. G1: control, G2: US, G3: Aza‐BD NPs, G4: Aza‐BD NPs + US, G5: Aza‐BD@PC NPs, and G6: Aza‐BD@PC NPs + US. Data are presented as mean ± SD. ^*^ *p* < 0.05, ^**^ *p* < 0.01, and ^***^ *p* < 0.001.

Furthermore, we investigated the combined antitumor effects of GT and SDT in vivo using a subcutaneous GL261 tumor‐bearing mouse model (**Figure** **7**a). The mice were randomly divided into six groups (n = 5 per group) as follows: 1) PBS, 2) US, 3) Aza‐BD NPs, 4) Aza‐BD NPs + US, 5) Aza‐BD@PC NPs, and 6) Aza‐BD@PC NPs + US. The volumes of the GL261 tumors distinctly increased in the control, US, and Aza‐BD NP groups, suggesting that US irradiation or Aza‐BD NPs alone had negligible effects on the inhibition of glioma growth (Figures 7b‐d). However, an apparent inhibition of glioma growth with a tumor inhibition rate (TIR) of 71.9% was observed in the Aza‐BD@PC NPs group, which can be solely attributed to the H_2_S‐induced GT effect. Similarly, the Aza‐BD NPs + US group showed an inhibitory effect on tumor propagation, with a TIR of 64.7%. Notably, the Aza‐BD@PC NPs + US group exhibited the most remarkable therapeutic effect, with a TIR of 85.9% (Figure 7e). Photographs of *ex vivo* gliomas from different groups revealed that the glioma volumes in the Aza‐BD@PC NPs + US group were substantially smaller than those in the other groups (Figure 7c). Similarly, the difference in the weights of the isolated gliomas in mice in each treatment group aligned with the results shown in Figure 7b,c (Figure 7f). Additionally, no evident changes in body weight were observed in any group throughout the treatment process, further indicating that the intravenous injection of Aza‐BD@PC NPs and US irradiation had no significant toxic effects on the mice (Figure 7g). Thus, the results confirmed that Aza‐BD@PC NPs demonstrated high therapeutic efficacy under US irradiation through the synergistic effect of GT and SDT.

Subsequently, hematoxylin and eosin (H&E) and terminal deoxynucleotidyl transferase uridine triphosphate nick end labeling staining was conducted to confirm the antitumor efficacy of the combined GT and SDT induced by Aza‐BD@PC NPs under US irradiation. The number of apoptotic and necrotic cells in the glioma tissues from the Aza‐BD@PC NPs + US group was higher than those in the other treatment groups. Additionally, Ki‐67 antibody staining images indicated that the Aza‐BD@PC NPs + US group displayed a remarkable suppressive effect on the proliferative activity of glioma cells (Figure 7h). Furthermore, DCFH‐DA and GPX4 staining was performed to elucidate the therapeutic mechanism of Aza‐BD@PC NPs plus US stimulation (Figure 7h). The DCFH‐DA staining images of the glioma slices in the Aza‐BD@PC NPs + US group displayed strong fluorescent signals, indicating the effective production of ^1^O_2_ at the tumor site by Aza‐BD@PC NPs under US irradiation. Additionally, GPX4 staining results revealed that GPX4 expression was remarkably reduced in the Aza‐BD@PC NPs + US group, confirming the induction of ferroptosis caused by the cooperative effect of GT and SDT. Furthermore, no pathological variations were observed in the H&E‐stained images of the major organs from all the treated groups, further verifying the high biocompatibility of the Aza‐BD@PC NPs (Figure S16, Supporting Information). These results demonstrate that Aza‐BD@PC NPs can significantly inhibit the proliferation of glioma cells under US irradiation through the synergistic effect of GT and SDT.

Furthermore, an orthotopic glioma model was established to evaluate the therapeutic efficacy of Aza‐BD@PC NPs under US irradiation. Mice bearing orthotopic gliomas were randomly divided into two groups (n = 3) as follows, Group 1: PBS; Group 2: Aza‐BD@PC NPs + US. An IVIS was used to monitor the growth of orthotopic gliomas in different treatment groups (Figure S17, Supporting Information). The results indicated that Aza‐BD@PC NPs significantly inhibited the growth of orthotopic gliomas under US irradiation compared to the control group, confirming the excellent therapeutic efficacy of Aza‐BD@PC NPs by the synergistic effect of GT and SDT under US irradiation.

## 3 Conclusion

We designed and engineered an intelligent nanoplatform, Aza‐BD@PC NPs, by assembling Aza‐BD using the biocompatible polymer PC‐modified DSPE‐PEG. After internalization by GBM cells, Aza‐BD@PC NPs selectively and effectively depleted intracellular Cys by the mild cleavage of S─N bonds. This process impeded GSH synthesis and produced H_2_S, which disrupted the intracellular redox balance and affected glycolytic pathways, thereby promoting ferroptosis and cell death. In addition, H_2_S caused DNA damage and inhibited the proliferation of GBM cells. Acidosis caused by H_2_S also provided a favorable acidic environment for ferroptosis initiation. Additionally, the liberated sonosensitizer generated a large amount of ^1^O_2_ under US excitation to achieve effective SDT. Importantly, mechanistic studies confirmed that Aza‐BD@PC administration and US stimulation could induce significant tumor suppression by regulating p53 signaling, apoptosis, and ferroptosis pathways. Therefore, this study not only offers a prospective approach to design innovative apoptosis and ferroptosis nanoinducers to effectively treat tumors but also provides an alternative avenue to overcome the barrier for effective GBM treatment.

## 4 Statistical Analysis

All data were presented as mean ± standard deviation and analyzed using GraphPad Prism 7.0. The significance was evaluated according to the unpaired Student's two‐sided test, two‐way ANOV A test, or one‐way ANOV A test. ^*^ *p* < 0.05, ^**^ *p* < 0.01, ^***^ *p* < 0.001, ^****^ *p* < 0.0001, ns: not significant.

## Conflict of Interest

The authors declare no conflict of interest.

## Supporting information

Supporting Information

## Data Availability

The data that support the findings of this study are available from the corresponding author upon reasonable request.
