# Supplementary material for: Self‐Assembled Aza‐Boron‐Dipyrromethene‐Based H2S Prodrug for Synergistic Ferroptosis‐Enabled Gas and Sonodynamic Tumor Therapies
Source: Adv Sci (Weinh). 2024 Jun 13;11(30):2309542. doi: 10.1002/advs.202309542 (PMC11321684; doi:10.1002/advs.202309542)
Supplement: Supplementary file 1 — Supporting Information [file ADVS-11-2309542-s001.pdf]

ADVANCED SCIENCE
Open Access

Supporting Information

for *Adv. Sci.*, DOI 10.1002/advs.202309542

Self-Assembled Aza-Boron-Dipyrromethene-Based H_2S Prodrug for Synergistic Ferroptosis-Enabled Gas and Sonodynamic Tumor Therapies

Jiajia Zhao, Erbao Bian, Renwu Zhang, Tao Xu, Yang Nie, Linqi Wang, Gui Jin, Han Xie, Huijing Xiang, Yu Chen* and Dejun Wu**

WILEY-VCH

ADVANCED SCIENCE
Open Access

Supporting Information

Self-Assembled Aza-Boron-Dipyrromethene-Based H_2S Prodrug for Synergistic Ferroptosis-Enabled Gas and Sonodynamic Tumor Therapies

Jiajia Zhao, Erbao Bian, Renwu Zhang, Tao Xu, Yang Nie, Linqi Wang, Gui Jin, Han Xie, Huijing Xiang*, Yu Chen*, and Dejun Wu*

J. Zhao, Prof. E. Bian, R. Zhang, Y. Nie, L. Wang, G. Jin, H. Xie, Prof. D. Wu

Department of Neurosurgery, The Second Affiliated Hospital of Anhui Medical University, Hefei, Anhui Province 230601, P. R. China. E-mail: wudj2023@163.com

Prof. Y. Chen, Prof. H. Xiang

School of Life Sciences, Shanghai University, Shanghai 200444, P. R. China. E-mail: chenyuedu@shu.edu.cn, xianghuijing@shu.edu.cn

Prof. Y. Chen
Oujiang Laboratory (Zhejiang Lab for Regenerative Medicine, Vision and Brain Health), Wenzhou Institute of Shanghai University, Wenzhou, Zhejiang 325088, P. R. China.

Prof. Y. Chen
Shanghai Institute of Materdicine, Shanghai 200051, P. R. China.

Prof. Tao Xu

Department of Neurosurgery, Changzheng Hospital, Naval Medical University, Shanghai 200003, China. P. R. China.

Experimental Procedures

Chemicals and materials.

Benzaldehyde, 4-aminoacetophenone and trifluoride diethyl etherate were acquired from Tokyo Chemical Industry (TCI, Japan). Ammonium acetate, chalcone, CH_2Cl_2, CH_3Cl, ethanol

(EtOH), methanol (MeOH), ethyl acetate (EtOAc), hydrochloric acid (HCl), hexane, sodium hydroxide (NaOH), potassium hydroxide (KOH), anhydrous tetrahydrofuran (THF), and phenyl chlorothionocarbonate were obtained from General-reagent® (China). Nitromethane and triethylamine were purchased from Adamas (Switzerland). DSPE-PEG-amine (DSPE-PEG-NH_2) was purchased from Shanghai Aladdin Biochemical Technology Co., Ltd (China). Fetal bovine serum (FBS), dulbecco's modified eagle medium (DMEM) and 0.25% trypsin-ethylene diamine tetraacetic acid (EDTA) solution were purchased from Thermo Fisher Scientific (USA). 1,3-diphenylisobenzofuran (DPBF), 9,10-anthracenediyl-bis(methylene) dimalonic acid (ABDA), 2,2,6,6-tetramethylpiperidine (TEMP), and 5,5'-dithio bis-(2-nitrobenzoic acid) (DTNB) were obtained from Aladdin (China). Washington state probe-1 (WSP-1) was obtained from Shanghai Maokang Biotechnology Co., Ltd (China). Annexin V-fluorescein isothiocyanate (FITC) apoptosis detection kit, calcein acetoxymethyl ester/propidium iodide (calcein-AM/PI), cell counting kit-8 (CCK-8), bisBenzimide H 33342 (Hoechst 33342), 2,7-dichlorodihydrofluorescein diacetate (DCFH-DA) assay kit, Cell Cycle and Apoptosis analysis kit, mitochondrial membrane potential assay kit with JC-1, adenosine triphosphate (ATP) assay kit, 2',7'-bis-(2-carboxyethyl)-5-(and-6)-carboxyfluorescein, acetoxymethyl ester (BCECF AM) and Lipid Peroxidation malondialdehyde (MDA) assay kit were obtained from Beyotime Biotechnology (China). CheKine™ Micro cysteine (Cys) assay kit, CheKine™ micro lactate assay kit and CheKine™ micro reduced glutathione (GSH) assay kit were obtained from Abbkine Biotechnology Co., Ltd (China). C11 BODIPY 581/591was bought from Thermo Fisher Scientific (USA).

Characterization.

^{1}H NMR and ^{13}C NMR spectra were recorded on Bruker Advance 300 and BBFO 400 spectrometers. Transmission electron microscopy (TEM) images were obtained using a JEM-1400F electron microscope at an accelerating voltage of 120 kV. Hydrodynamic diameters and zeta potential were measured by a Zeta sizer Nano series (Nano ZS90, Malvern Instrument Ltd.). Fourier transformed infrared (FTIR) spectra were recorded by a VERTEX70V FTIR spectrometer (Bruker, Germany). UV-vis absorption spectra were measured on a U-3900H UV-vis spectrometer (HITACHI, Japan). *In vitro* singlet oxygen ($^{1}O_2$) generation was measured by an EMXplus electron spin resonance (ESR) spectrometer (Bruker, Germany). Confocal images were obtained by a confocal laser scanning microscope (CLSM 710; Carl Zeiss, Germany). Flow cytometric analysis for apoptosis, cellular uptake, intracellular reactive oxygen species

(ROS) generation, and hydrogen sulfide (H_2S) generation were performed by flow cytometry (Beckman, USA). In vivo biodistribution was analyzed by an in vivo fluorescence system (PerkinElmer, USA). Ultrasound (US) therapeutic apparatus (Encore Medical International, Inc.) was used as an irradiation source for implementing sonodynamic therapy (SDT).

Synthesis of the amino chalcone.

To an aqueous solution of NaOH (1.20 g, 30 mmol), 4-aminoacetophenone (1.35 g, 10 mmol) was added and stirred for 10 min. Then, benzaldehyde (1.27 g, 12 mmol) was added dropwise to the stirred solution, then the reaction mixture was stirred at 90 °C and refluxed for 6 h. The resulting precipitate was filtered, washed with ice-cold water, and dried to obtain the corresponding chalcone as a yellow solid. The mixture was further washed with water and extracted with ethyl acetate. After the solvent was removed, the residue was obtained, which was subsequently purified by column chromatography using silica gel. The mixture of ethyl acetate and hexane at a ratio of 2:8 was chromatographed to obtain product **1** (1.50 g, 6.7 mmol). ^{1}H NMR (500 MHz, Chloroform-d) δ 7.97 – 7.91 (m, 2H), 7.91 – 7.81 (m, 1H), 7.78 (d, J = 15.7 Hz, 1H), 7.66 – 7.61 (m, 2H), 7.56 (s, 1H), 7.55 – 7.47 (m, 1H), 7.47 – 7.28 (m, 6H), 7.06 – 6.94 (m, 1H), 6.74 – 6.62 (m, 3H), 5.40 – 5.32 (m, 1H), 5.30 (s, 3H), 4.12 (q, J = 7.1 Hz, 2H), 2.25 – 2.19 (m, 1H), 2.01 (q, J = 6.4 Hz, 3H), 1.63 (p, J = 7.6 Hz, 3H), 1.33 (s, 3H), 1.30 – 1.21 (m, 18H), 0.91 – 0.82 (m, 3H).

Synthesis of the nitromethane adduct.

Chalcone (1.3 g, 5.76 mmol) was dissolved in MeOH (80 mL), activated KOH (0.30 g, 5.25 mmol) and nitromethane (2 mL) was then added to the above solution and refluxed for 15 h. The resulting mixture was washed with water and then extracted with chloroform. After the solvent is removed, the residue was obtained, which was subsequently purified by column chromatography using silica gel. The mixture of ethyl acetate and hexane at a ratio of 2:8 was chromatographed to obtain product **2** (0.70 g, 2.45 mmol). ^{1}H NMR (400 MHz, Chloroform-d) δ 12.57 (d, J = 2.0 Hz, 1H), 12.55 – 12.47 (m, 4H), 12.14 – 11.96 (m, 9H), 11.48 – 11.32 (m, 5H), 9.60 (dd, J = 12.5, 6.2 Hz, 2H), 9.42 (dd, J = 12.5, 8.6 Hz, 2H), 9.06 (s, 3H), 8.97 (dtd, J = 14.4, 8.3, 7.2, 4.1 Hz, 3H), 8.18 – 7.97 (m, 3H), 7.26 (s, 3H).

Synthesis of azadipyrromethene.

Product **2** (0.70 g, 2.45 mmol) and ammonium acetate (7.6 g, 95 mmol) were dissolved in EtOH (20 mL) and refluxed for 48 h. The solution was filtered, washed with cold EtOH, and then dried. The resulting solution was washed with water and then extracted with ethyl acetate. After the solvent was removed, the residue was obtained, which was subsequently purified by column chromatography using silica gel. The mixture of MeOH and CH_2Cl_2 at a ratio of 1:100 was eluted to obtained product **3** (0.22 g, 0.45 mmol). ^{1}H NMR (500 MHz, Chloroform-d) δ 8.82 (s, 1H), 3.61 – 3.53 (m, 1H), 3.20 (p, J = 7.6 Hz, 1H), 3.14 (s, 3H), 2.87 (s, 1H), 2.87 – 2.77 (m, 12H), 2.47 – 2.37 (m, 3H).

Synthesis of Aza-BD probe.

Product **3** (0.22 g, 0.45 mmol) was dissolved in dry dichloromethane (80 mL) and treated with triethylamine (0.8 mL, 4.6 mmol). The mixture was stirred at 30°C for 10 min. Next, boron trifluoride diethyl etherate (1 mL, 8.13 mmol) was added and the reaction mixture was stirred at 25°C for 15 h. The solvent was then evaporated, and the resulting product was washed with water (2 × 50 mL) and extracted with chloroform. After the solvent was removed, the residue was obtained, which was subsequently purified by column chromatography using silica gel. The mixture of ethyl acetate and hexane at a 1:1 ratio was eluted to obtain the **probe 1 (0.13 g, 0.25 mmol)**. ^{1}H NMR (400 MHz, Chloroform-d) δ 7.92 – 7.79 (m, 2H), 7.26 (d, J = 7.0 Hz, 1H), 7.23 – 7.17 (m, 1H), 6.85 (s, 1H), 6.55 (d, J = 8.7 Hz, 1H), 1.36 (s, 7H), 1.06 (s, 3H), -0.13 (s, 1H). ^{13}C NMR (151 MHz, $CDCl_3$) δ 132.97, 101.89, 77.34, 77.16, 76.59, 74.58, 69.66, 68.29, 66.87, 62.74, 24.56, 24.35, 24.14, 17.73, -18.77, -20.89, -23.10, -23.15, -23.29, -23.45, -23.70, -27.95. MS (TOF ES$^+$) Calcd. for $C_{32}H_{24}BF_2N_5$ (M+H$^+$):526.2129, Found: 526.2037.

Synthesis of DSPE-PEG-PC.

Phenyl chlorothionocarbonate (17.5 mg, 0.05 mmol) was slowly added to anhydrous THF (3 mL) solution containing DSPE-PEG-NH_2 (50 mg, 0.05 mmol). The reaction mixture was stirred at room temperature for 5 h under nitrogen. After the solvent was removed, the product was obtained. ^{1}H NMR (600 MHz, Chloroform-d) δ 7.47 – 7.40 (m, 9H), 7.35 – 7.29 (m, 5H), 7.12 (dd, J = 8.0, 1.5 Hz, 9H), 3.63 (s, 26H), 1.28 (s, 2H), 1.25 (s, 7H), 0.87 (t, J = 6.9 Hz, 1H). ^{13}C NMR (151 MHz, $CDCl_3$) δ 149.19, 142.08, 131.77, 129.13, 128.80, 128.41, 114.67, 77.16, 76.95, 76.74, 29.63. MS (TOF ES$^+$) Calcd for $C_{137}H_{263}N_2O_{54}PS$ (M+H$^+$): 2863.735, Found: 2863.059.

Synthesis of Aza-BD@PC nanoparticles (NPs).

Aza-BD (5 mg) and DSPE-PEG-PC (50 mg) were added into chloroform (50 mL), and the mixture was stirred at room temperature for 24 h. The solvent was removed by a rotary evaporator. Ultrapure water (10 mL) was then added to the drying mixture to obtain an Aza-BD nanoparticle solution. The solution was purified using a 0.2 μm syringe filter to obtain the final product.

Evaluation of in vitro $^1O_2$ generation.

$^1O_2$ generation was detected by using DPBF as a $^1O_2$ indicator. In a typical experiment, EtOH solution (10 μL) containing DPBF (10 mmol L^{-1}) was added to 2 mL of PBS solution containing Aza-BD NPs or Aza-BD@PC NPs. The solutions were exposed to US irradiation (1.0 W cm^{-2}) for 5 min, respectively. The UV-vis absorption spectra of the above solutions were recorded every minute. The same method was used for ABDA detection.

For ESR spectroscopy measurements, $^1O_2$ generation was detected using TEMP as a $^1O_2$ trapping agent. The various treatment groups were set up as follows: (1) US, (2) Aza-BD NPs, (3) Aza-BD NPs + US, (4) Aza-BD@PC NPs, and (5) Aza-BD@PC NPs + US. After various treatments, the aqueous solution (180 μL) from each treatment group was mixed with TEMP solution (20 μL, 1 M) in a vial. Then, each sample was transferred to a quartz capillary tube, and the ESR signal was recorded at room temperature using an ESR spectrometer.

Evaluation of in vitro Cys depletion.

Cys depletion was detected by using DTNB as a Cys indicator. Cys (20 μmol L^{-1}) was added to different concentrations of Aza-BD@PC NPs aqueous solution. PBS solution (50 μL) containing DTNB (1.5 mg mL^{-1}) was added to the above solutions, and the mixture was transferred to a cuvette and left stand for 10 min. The UV-vis absorption spectra of the above solutions were recorded.

Evaluation of in vitro H_2S generation.

H_2S generation was detected by using WSP-1 as an H_2S indicator. Cys (20 μmol L^{-1}) was added to various concentrations of Aza-BD@PC NPs aqueous solution. WSP-1 probe (10 μM) was added to the above solutions, and the mixture was placed at 37°C 30 min. The fluorescence

intensities of the above solutions at 515 nm were detected at an excitation wavelength of 465 nm. For lead acetate test paper detection. Cys (20 μmol L^{-1}) was added to diverse concentrations of Aza-BD@PC NPs aqueous solution. The above solutions were added dropwise to a moistened lead acetate test paper. The test paper of each treatment group was placed at room temperature for 24 h to observe the color change.

Cell culture.

The mouse glioma cancer cell line (GL261 cells) was obtained from the Shanghai Institute of Cells, Chinese Academy of Sciences. The cells were cultured in DMEM medium (Gibco) supplemented with 10% fetal bovine serum (FBS, Gibco) and 1% penicillin-streptomycin (Invitrogen). The cells were maintained at 37°C in a 5% CO_2 environment.

In vitro cytotoxicity of Aza-BD@PC NPs against 3T3 and GL261 cells under US irradiation.

To assess the cytotoxicity of Aza-BD NPs and Aza-BD@PC NPs, 3T3 and GL261 cells (5 × 10^3 cells/well) were seeded in 96-well plates and cultured overnight. The cells were treated with Aza-BD NPs and Aza-BD@PC NPs at different concentrations and incubated for 24 h. Then, the cells were washed three times with PBS, and the cell viabilities of Aza-BD NPs and Aza-BD@PC NPs were detected using a standard CCK-8 assay. To evaluate the cell viabilities of Aza-BD NPs and Aza-BD@PC NPs under US irradiation, GL261 cells were seeded in 96-well plates and incubated overnight. The cells were treated with Aza-BD NPs and Aza-BD@PC NPs at different concentrations and incubated for 12 h. US irradiation was then applied at a power density of 1.0 W cm^{-2} for 5 min by an ultrasonic apparatus. The cell viabilities after various treatments were determined by a standard CCK-8 assay.

Cellular uptake of Aza-BD@PC NPs.

GL261 cells were seeded in CLSM-specific dishes and incubated for 24 h. The cells were then incubated with FITC-labeled Aza-BD@PC NPs (50 ppm) for various time points (0, 1, 2, 4, and 8 h). After washed with PBS for three times, the cells were stained with Hoechst 33342 (1 μM) for 20 min and imaged by CLSM observation. GL261 cells were seeded in 6-well culture plates and cultured for 24 h. The cells were then incubated with FITC-labeled Aza-BD@PC NPs (50 ppm) for various time points (0, 1, 2, 4, and 8 h). After washed three times with PBS,

the cells were collected by trypsinization and centrifugation, dispersed in PBS, and then analyzed by flow cytometry.

Intracellular ROS generation evaluation.

GL261 cells were seeded in CLSM-specific culture dishes and incubated for 24 h. The cells then received various treatments, including PBS, US, Aza-BD NPs (50 ppm), Aza-BD NPs (50 ppm) + US, Aza-BD@PC NPs (50 ppm), and Aza-BD@PC NPs (50 ppm) + US. US irradiation was then applied at a power density of 1.0 W cm^{-2} for 5 min by an ultrasonic apparatus. After various treatments, DCFH-DA was added and incubated for 30 min. Finally, the cells were washed three times with PBS, and observed by CLSM. GL261 cells were seeded in 6-well plates and cultured for 24 h. The cells then received various treatments, including PBS, US, Aza-BD NPs (50 ppm), Aza-BD NPs (50 ppm) + US, Aza-BD@PC NPs (50 ppm), and Aza-BD@PC NPs (50 ppm) + US. US irradiation was then applied at a power density of 1.0 W cm^{-2} for 5 min by an ultrasonic apparatus. After various treatments, DCFH-DA was added and incubated for 30 min. After washed with PBS for three times, the cells were collected by trypsinization and centrifugation, then dispersed in PBS, and analyzed by flow cytometry.

Intracellular H_2S generation evaluation.

GL261 cells were seeded in CLSM-specific dishes and incubated for 24 h. The cells were incubated with Aza-BD@PC NPs at different concentrations for 6 h. After various treatments, the cells were washed with PBS for three times, WSP-1 was added and incubated for 1 h, and then observed by CLSM. GL261 cells were seeded in 6-well culture plates and cultured for 24 h. The cells received various treatments, including PBS, US, Aza-BD NPs (50 ppm), Aza-BD NPs (50 ppm) + US, Aza-BD@PC NPs (50 ppm), and Aza-BD@PC NPs (50 ppm) + US. US irradiation was then applied at a power density of 1.0 W cm^{-2} for 5 min by an ultrasonic apparatus. After various treatments, the cells were washed with PBS for three times, WSP-1 was added and incubated for 1 h. After washed with PBS for three times, the cells were collected by trypsinization and centrifugation, dispersed in PBS, and then analyzed by flow cytometry.

In vitro live/dead staining measurement.

GL261 cells were seeded in CLSM-specific dishes and incubated for 24 h. The cells received various treatments, including PBS, US, Aza-BD NPs (50 ppm), Aza-BD NPs (50 ppm) + US,

Aza-BD@PC NPs (50 ppm), and Aza-BD@PC NPs (50 ppm) + US. US irradiation was then applied at a power density of 1.0 W cm^{-2} for 5 min by an ultrasonic apparatus. After washed with PBS for three times, calcein-AM and PI were added to the CLSM dishes to label live and dead cells, respectively. After staining for 30 min, the cells were washed with PBS for twice and imaged by CLSM observation.

In vitro cellular apoptosis assessment.

GL261 cells were seeded in 6-well plates and cultured for 24 h. The cells then received various treatments, including PBS, US, Aza-BD NPs (50 ppm), Aza-BD NPs (50 ppm) + US, Aza-BD@PC NPs (50 ppm), and Aza-BD@PC NPs (50 ppm) + US. US irradiation was then applied at a power density of 1.0 W cm^{-2} for 5 min by an ultrasonic apparatus. After washed with PBS for three times, the cells were collected by trypsinization and centrifugation, and then dispersed in PBS. The cells were labeled with Annexin V-FITC and PI, and then analyzed by flow cytometry.

Intracellular Cys level detection.

GL261 cells were seeded in 6-well plates and cultured for 24 h. The cells then received various treatments, including PBS, US, Aza-BD NPs (50 ppm), Aza-BD NPs (50 ppm) + US, Aza-BD@PC NPs (50 ppm), and Aza-BD@PC NPs (50 ppm) + US. US irradiation was then applied at a power density of 1.0 W cm^{-2} for 5 min by an ultrasonic apparatus. After washed with PBS for three times, the cells were collected by trypsinization and centrifugation. The intracellular Cys content in different treatment groups was determined by the Cys detection kit.

Cell cycle detection.

GL261 cells were seeded in 6-well plates and cultured at 37 °C for 24 h. The cells then received different treatments, including PBS, US, Aza-BD NPs (50 ppm), Aza-BD NPs (50 ppm) + US, Aza-BD@PC NPs (50 ppm), and Aza-BD@PC NPs (50 ppm) + US. US irradiation was then applied at a power density of 1.0 W cm^{-2} for 5 min by an ultrasonic apparatus. After washed with PBS for three times, the cells were collected by trypsinization and centrifugation, and then dispersed in PBS. The cells were labeled with PI, and then analyzed by flow cytometry.

Intracellular JC-1 measurement.

GL261 cells were seeded in CLSM-specific dishes and cultured at 37 °C for 24 h. The cells then received different treatments, including PBS, US, Aza-BD NPs (50 ppm), Aza-BD NPs (50 ppm) + US, Aza-BD@PC NPs (50 ppm), and Aza-BD@PC NPs (50 ppm) + US. US irradiation was then applied at a power density of 1.0 W cm^{-2} for 5 min by an ultrasonic apparatus. After various treatments, the cells were washed with PBS for three times, and incubated with JC-1 (10 μM) for 30 min. Confocal images were recorded by CLSM at excitation wavelengths of 543 and 488 nm, respectively.

Intracellular ATP content detection.

GL261 cells were seeded in 6-well plates and cultured for 24 h. Then, the cells received various treatments, including PBS, US, Aza-BD NPs (50 ppm), Aza-BD NPs (50 ppm) + US, Aza-BD@PC NPs (50 ppm), and Aza-BD@PC NPs (50 ppm) + US. US irradiation was then applied at a power density of 1.0 W cm^{-2} for 5 min by an ultrasonic apparatus. After washed with PBS for three times, the cells were collected by trypsinization and centrifugation. The intracellular ATP content in different treatment groups was determined by the ATP detection kit.

Intracellular lactic acid level detection.

GL261 cells were seeded in 6-well plates and cultured for 24 h. Then, the cells received various treatments, including PBS, US, Aza-BD NPs (50 ppm), Aza-BD NPs (50 ppm) + US, Aza-BD@PC NPs (50 ppm), and Aza-BD@PC NPs (50 ppm) + US. US irradiation was then applied at a power density of 1.0 W cm^{-2} for 5 min by an ultrasonic apparatus. After washed with PBS for three times, the cells were collected by trypsinization and centrifugation. The lactic acid detection kit was used to determine the intracellular lactic acid content in different treatment groups.

In vitro cellular pH assessment.

GL261 cells were seeded in CLSM- specific dishes and cultured at 37 °C for 24 h. The cells then received different treatments, including PBS, US, Aza-BD NPs (50 ppm), Aza-BD NPs (50 ppm) + US, Aza-BD@PC NPs (50 ppm), and Aza-BD@PC NPs (50 ppm) + US. US irradiation was then applied at a power density of 1.0 W cm^{-2} for 5 min by an ultrasonic

apparatus. After various treatments, the cells were washed with PBS for three times, and incubated with BCECF-AM (5 μM) for 30 min. Confocal images were recorded by CLSM at excitation wavelengths of 488 and 405 nm, respectively.

Intracellular GSH content detection.

GL261 cells were seeded in 6-well plates and cultured for 24 h. Then, the cells received various treatments, including PBS, US, Aza-BD NPs (50 ppm), Aza-BD NPs (50 ppm) + US, Aza-BD@PC NPs (50 ppm), and Aza-BD@PC NPs (50 ppm) + US. US irradiation was then applied at a power density of 1.0 W cm^{-2} for 5 min by an ultrasonic apparatus. After washed with PBS for three times, the cells were collected by trypsinization and centrifugation. The GSH assay kit was used to determine the intracellular GSH level in different treatment groups.

Intracellular MDA content detection.

GL261 cells were seeded in 6-well plates and cultured for 24 h. Then, the cells received various treatments, including PBS, US, Aza-BD NPs (50 ppm), Aza-BD NPs (50 ppm) + US, Aza-BD@PC NPs (50 ppm), and Aza-BD@PC NPs (50 ppm) + US. US irradiation was then applied at a power density of 1.0 W cm^{-2} for 5 min by an ultrasonic apparatus. After washed with PBS for three times, the cells were collected by trypsinization and centrifugation. The intracellular MDA content in each treatment group was determined by the MDA detection kit.

Intracellular LPO measurement.

GL261 cells were seeded in CLSM-specific dishes and cultured at 37 °C for 24 h. The cells then received different treatments, including PBS, US, Aza-BD NPs (50 ppm), Aza-BD NPs (50 ppm) + US, Aza-BD@PC NPs (50 ppm), and Aza-BD@PC NPs (50 ppm) + US. US irradiation was then applied at a power density of 1.0 W cm^{-2} for 5 min by an ultrasonic apparatus. After various treatments, the cells were washed with PBS for three times, and incubated with C11-BODIPY (10 μM) for 30 min. Confocal images were recorded by CLSM at excitation wavelengths of 543 and 488 nm, respectively.

Western blot analysis.

GL261 cells were seeded in 6-well plates and cultured for 24 h. Then, the cells received various treatments, including PBS, US, Aza-BD NPs (50 ppm), Aza-BD NPs (50 ppm) + US, Aza-BD@PC NPs (50 ppm), and Aza-BD@PC NPs (50 ppm) + US. US irradiation was then applied at a power density of 1.0 W cm^{-2} for 5 min by an ultrasonic apparatus. After incubation for 8 h, the cells were washed with cold PBS, collected by centrifugation, and completely lysed on ice using lysis buffer for 30-60 min. The proteins in the supernatant were harvested by centrifugation at 12000 rpm for 15 min, and the concentrations of proteins were determined by a bicinchoninic acid (BCA) protein assay kit (Beyotime Biotechnology, China). Equivalent proteins were loaded on sodium dodecyl sulfate polyacrylamide gel electrophoresis (SDS-PAGE, Beyotime Biotechnology, China), and transferred to 0.45 μm polyvinylidene fluoride (PVDF, IPVH00010, Millipore, USA) membranes. The PVDF membranes were then incubated overnight in primary antibodies (GPX4 and GAPDH: ABclonal, China) with slight shaking at 4 °C. The target proteins were incubated with horseradish peroxidase-conjugated goat-anti-Rabbit or goat-anti-Mouse secondary antibodies at room temperature for 1 h. Finally, the protein bands were visualized using a ChemiScope Touch chemiluminescence and fluorescence system (ChemiScope 6100, CLINX, China).

In vivo toxicity evaluation.

The animal experiment was approved by the Ethic Committee of Shanghai University (Approval No. ECSHU-2022-050). Fifteen healthy Kunming mice were randomly divided into 3 groups (n = 5) and received various treatments, including PBS, Aza-BD@PC NPs (5 mg kg^{-1}), and Aza-BD@PC NPs (10 mg kg^{-1}). After intravenous injection for 30 days, the mice in each treatment group were sacrificed, and the major organs and blood samples of the representative mice were collected for histological analysis and blood examination, respectively.

In vivo fluorescence imaging.

To investigate the biodistribution behavior of Aza-BD@PC NPs, GL261 tumor-bearing mice were injected with Cy5.5 labeled Aza-BD@PC NPs (5 mg kg^{-1}) through the tail vein. The mice were imaged using an in vivo fluorescence imaging system at different incubation intervals (0, 2, 4, 6, 8, 12, and 24 h).

In vivo therapeutic assessment.

GL261 cells (10^6 cells/mouse) were dispersed in PBS (100 μL) and injected into the right hind leg of nude mice to establish GL261 tumor xenografts. GL261 tumor-bearing mice with uniform tumor sizes were randomly divided into 6 groups (n = 5), and subjected to various treatments as follows: (1) PBS, (2) US (1.5 W cm^{-2}, 5 min), (3) Aza-BD NPs (5 mg kg^{-1}, 100 μL), (4) Aza-BD NPs (5 mg kg^{-1}, 100 μL) + US (1.5 W cm^{-2}, 5 min), (5) Aza-BD@PC NPs (5 mg kg^{-1}, 100 μL), and (6) Aza-BD@PC NPs (5 mg kg^{-1}, 100 μL) + US (1.5 W cm^{-2}, 5 min). After various treatments, the body weights and tumor volumes of the mice in each treatment group were recorded every other day for 14 days. At the end of the observation period, the major organs and tumor tissues of the representative mice in all treatment groups were collected for histological analysis.

Histological analysis.

At the end of the observation period, the tumor tissues of the representative mice were harvested for hematoxylin-eosin (H&E), TdT-mediated dUTP Nick-End Labeling (TUNEL), and Ki-67 staining to assess the therapeutic efficacy of Aza-BD@PC NPs under US irradiation. GPX4 staining was conducted to assess the expression levels of GPX4 in tumor tissues from different treatment groups. DCFH-DA staining was perofrmed to evaluate the ROS levels in tumor tissues from each treatment group.

Orthotopic glioma model establishment.

Luminescence-labeled GL261 cells were harvested by trypsinization, washed with PBS, and resuspended (10^6 cells/mouse) in PBS for implantation into the right caudate nucleus of mouse brains. Mice were anesthetized by intraperitoneal injection of 0.8% pentobarbital sodium (20 mL kg^{-1}) and the head was fixed to a brain stereotactic instrument in a prone position. After disinfecting the skin on the top of the head with 75% ethanol, a vertical incision was made behind the ocular fissure in the midline of the skull to expose the anterior fontanelle. A sagittal incision was made through the skin overlying the calvarium, and a small dental drill was used to make a hole in the exposed cranium, 1 mm anterior and 2.5 mm lateral to the right side of the sagittal suture. Luminescence-labeled GL261 cell suspension was injected into the white matter area vertically through the hole. The injection was performed over a 2 min period, and the needle was withdrawn over another 2 min. Mice bearing orthotopic gliomas were randomly divided into two groups (n =3) as follows, Group 1: PBS; Group 2: Aza-BD@PC NPs + US. An IVIS was used to monitor the growth of orthotopic gliomas in different treatment groups.

Supplementary figures

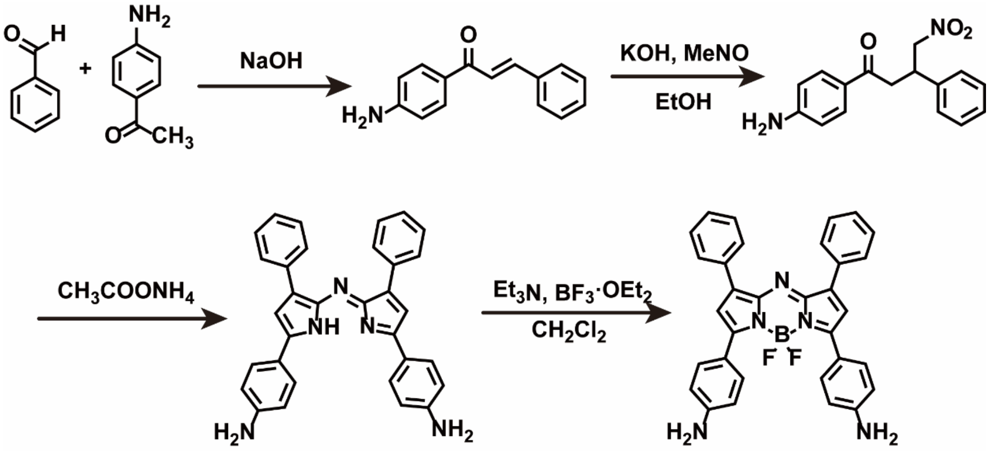

Scheme S1. Synthetic route of Aza-BD.

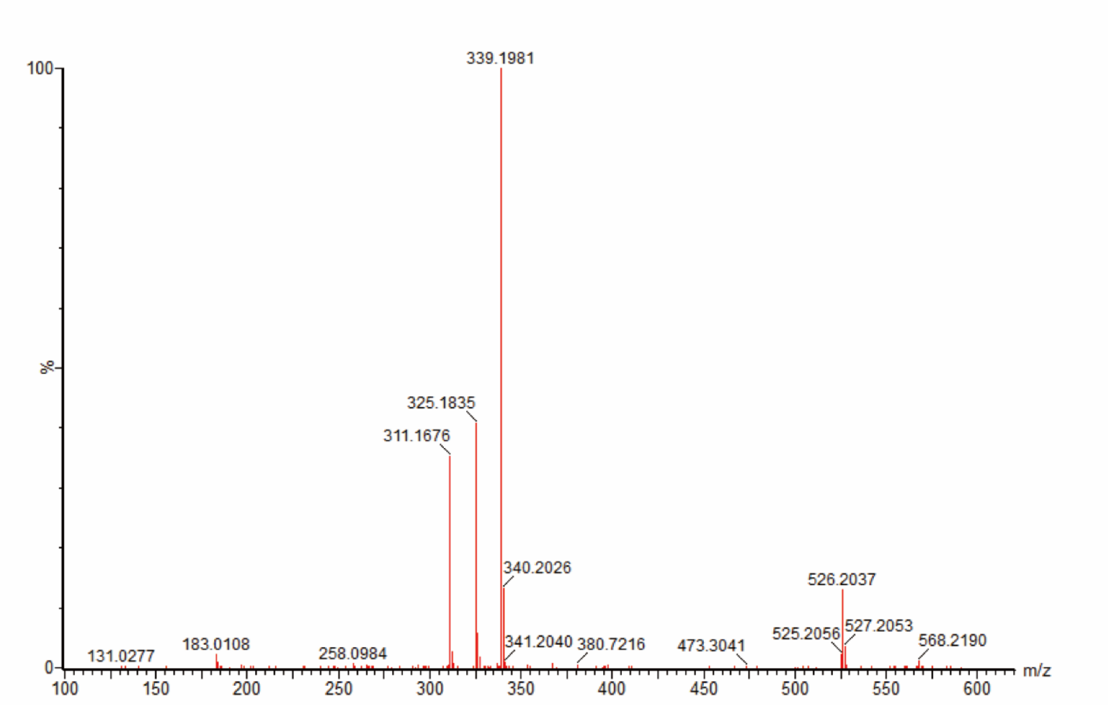

Figure S1. TOF-MS spectrum of Aza-BD.

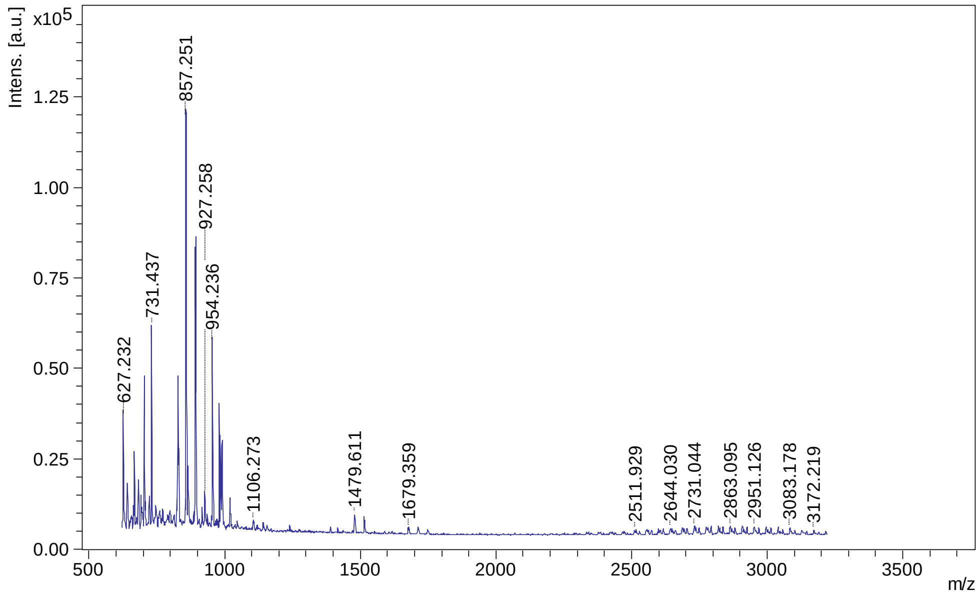

Figure S2. MALDI-TOF-MS spectrum of DSPE-PEG-PC.

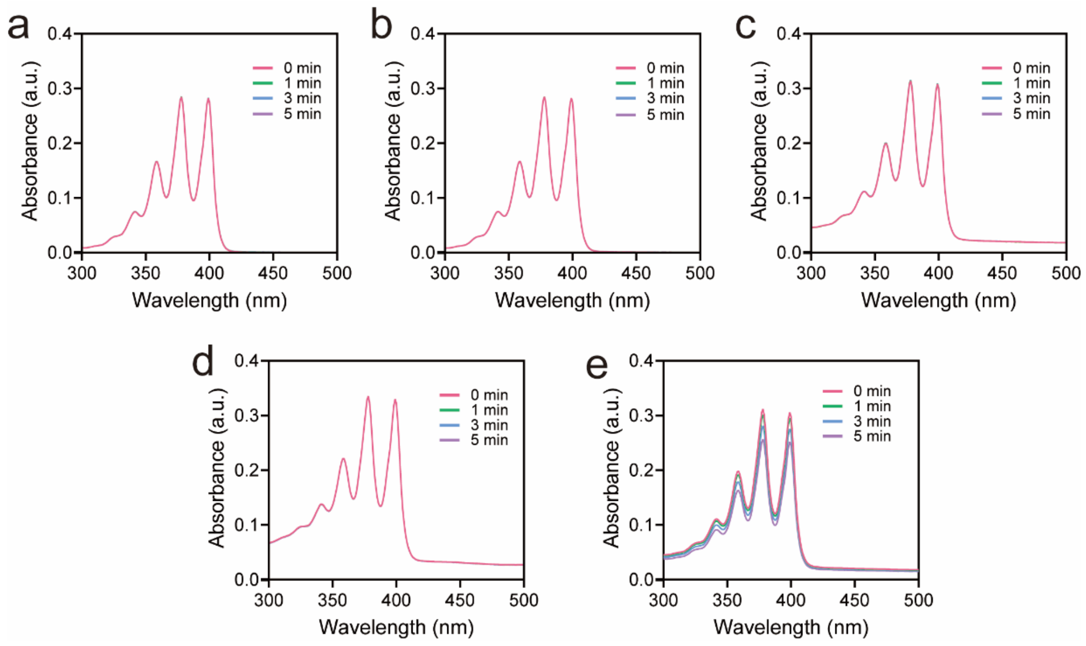

Figure S3. UV-vis absorption spectra of ABDA aqueous solutions containing a) PBS, b) PBS under US irradiation, c) Aza-BD NPs, d) Aza-BD@PC NPs, and e) Aza-BD@PC NPs under US irradiation.

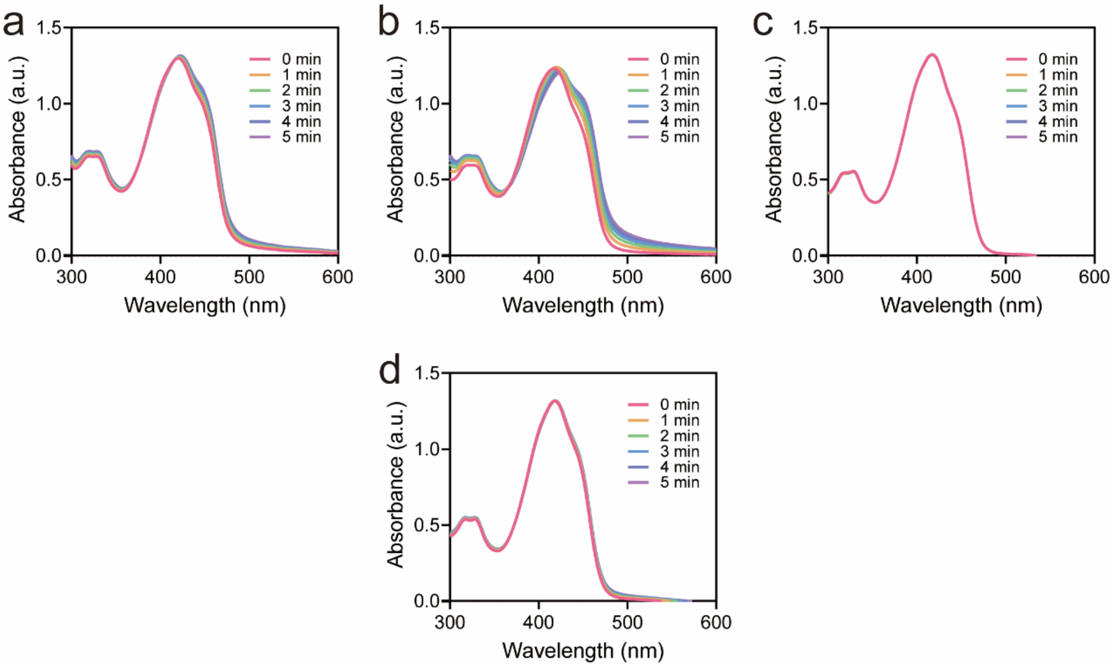

Figure S4. UV-vis absorption spectra of DPBF aqueous solutions containing a) PBS, b) PBS under US irradiation, c) Aza-BD NPs, and d) Aza-BD@PC NPs.

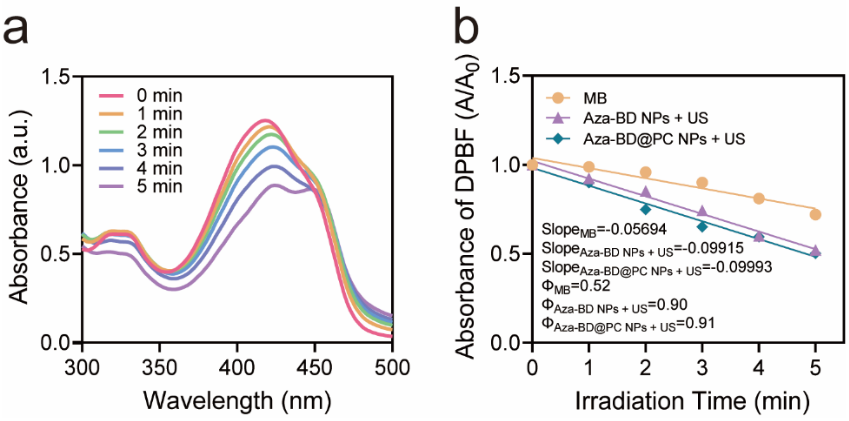

Figure S5. (a) UV-vis absorption spectra of DPBF aqueous solution containing MB under US irradiation for 5 min. (b) Degradation kinetics of DPBF by MB, Aza-BD NPs and Aza-BD@PC NPs under US irradiation for 5 min (n = 3). Data are represented as mean ± SD.

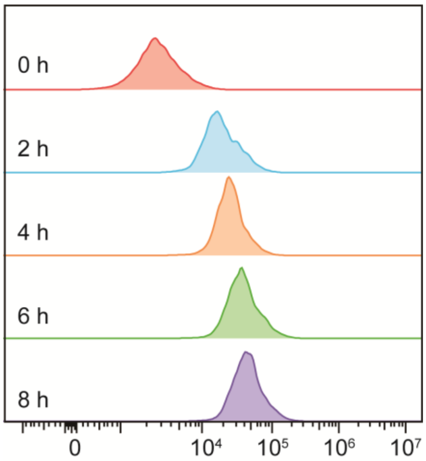

Figure S6. Flow cytometric analysis of the intracellular uptake of FITC-labeled Aza-BD@PC-FITC NPs at varying incubation time.

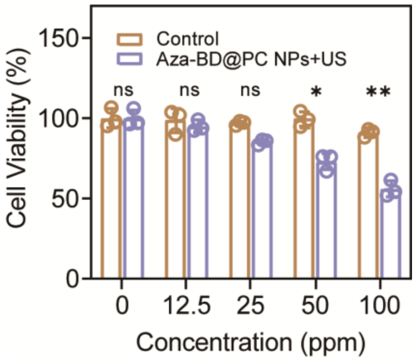

Figure S7. Cell viabilities of 4T1 cells treated with different doses of Aza-BD@PC NPs under US irradiation (n = 5). Data are represented as mean ± SD and analyzed by one-way ANOVA. ns: no statistical difference, *$p < 0.05$, **$p < 0.01$, and ***$p < 0.001$.

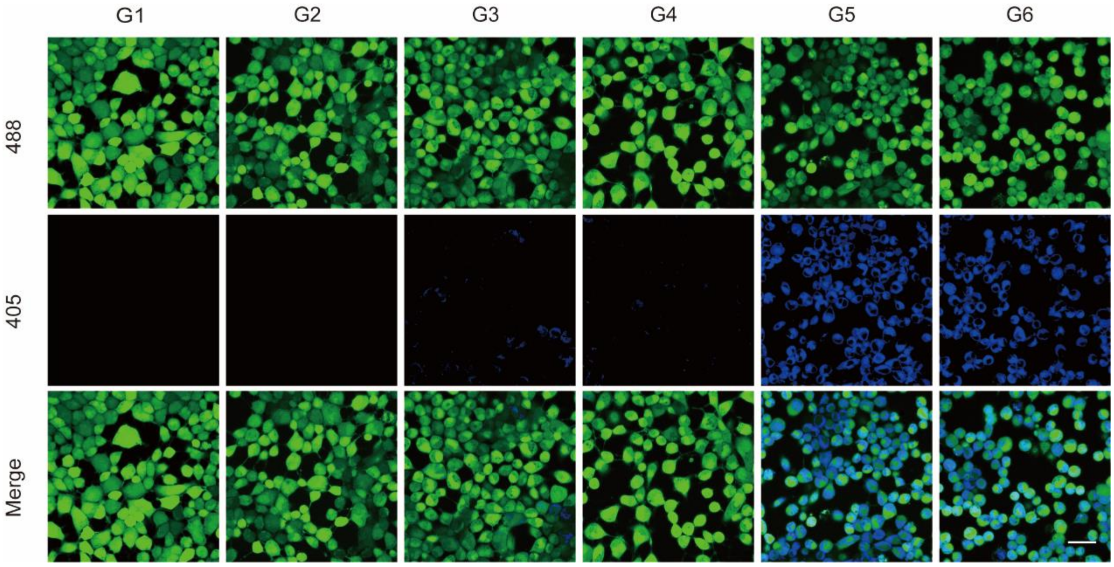

Figure S8. Confocal images of GL261 cells after various treatments using a BCECF probe (scale bar: 50 μm). G1: Control, G2: US, G3: Aza-BD NPs G4: Aza-BD NPs + US, G5: Aza-BD@PC NPs, G6: Aza-BD@PC NPs + US.

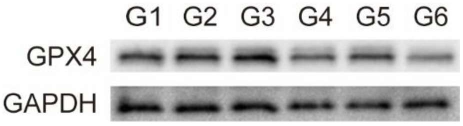

Figure S9. Western blotting of the GPX4 level in GL261 cells after various treatments. G1: Control, G2: US, G3: Aza-BD NPs G4: Aza-BD NPs + US, G5: Aza-BD@PC NPs, G6: Aza-BD@PC NPs + US.

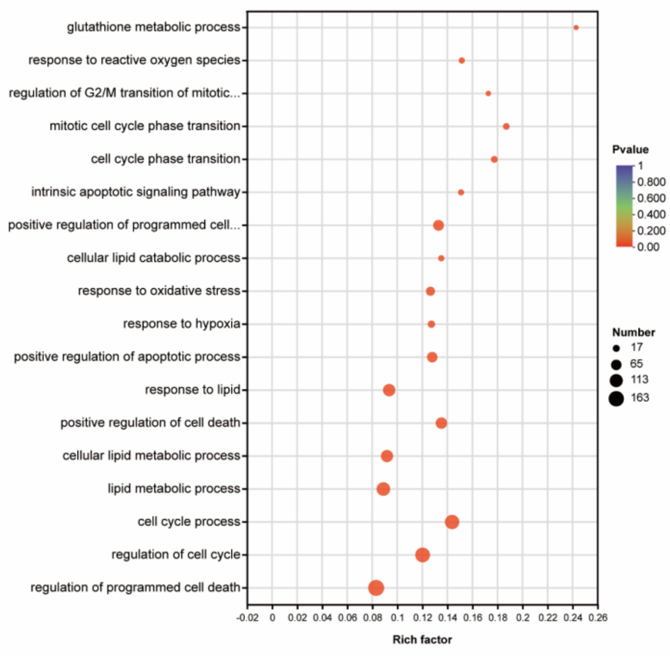

Figure S10. Bubble diagram of the differentially expressed genes (DEGs) enriched in the gene ontology (GO) pathway.

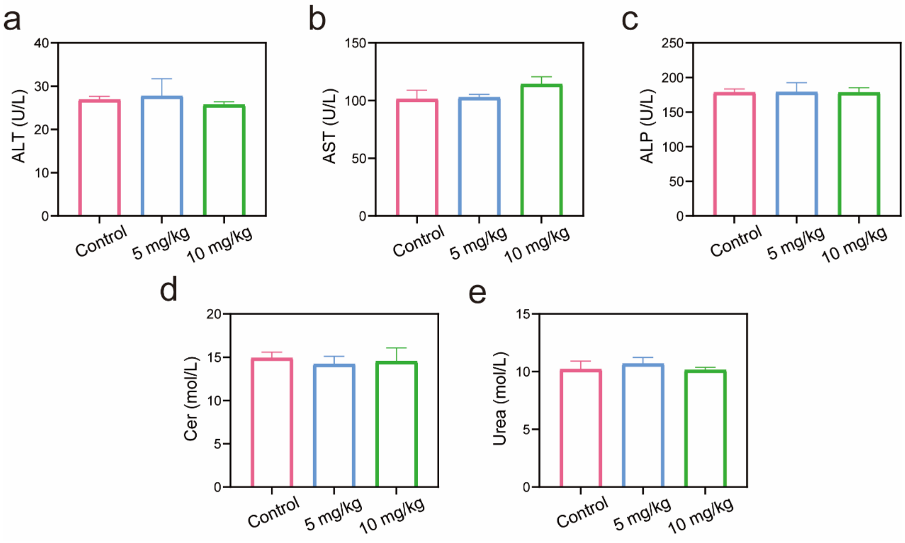

Figure S11. Biochemical parameters of the Kunming mice after intravenous injection of PBS (control) and Aza-BD@PC NPs at different concentrations, including (a) alanine aminotransferase (ALT), (b) ceramic oxalacetic transaminase (AST), and (c) alkaline phosphatase (ALP), as well as (d) creatinine (Cre) and (e) urea (Urea) (n =5). Data are represented as mean ± SD.

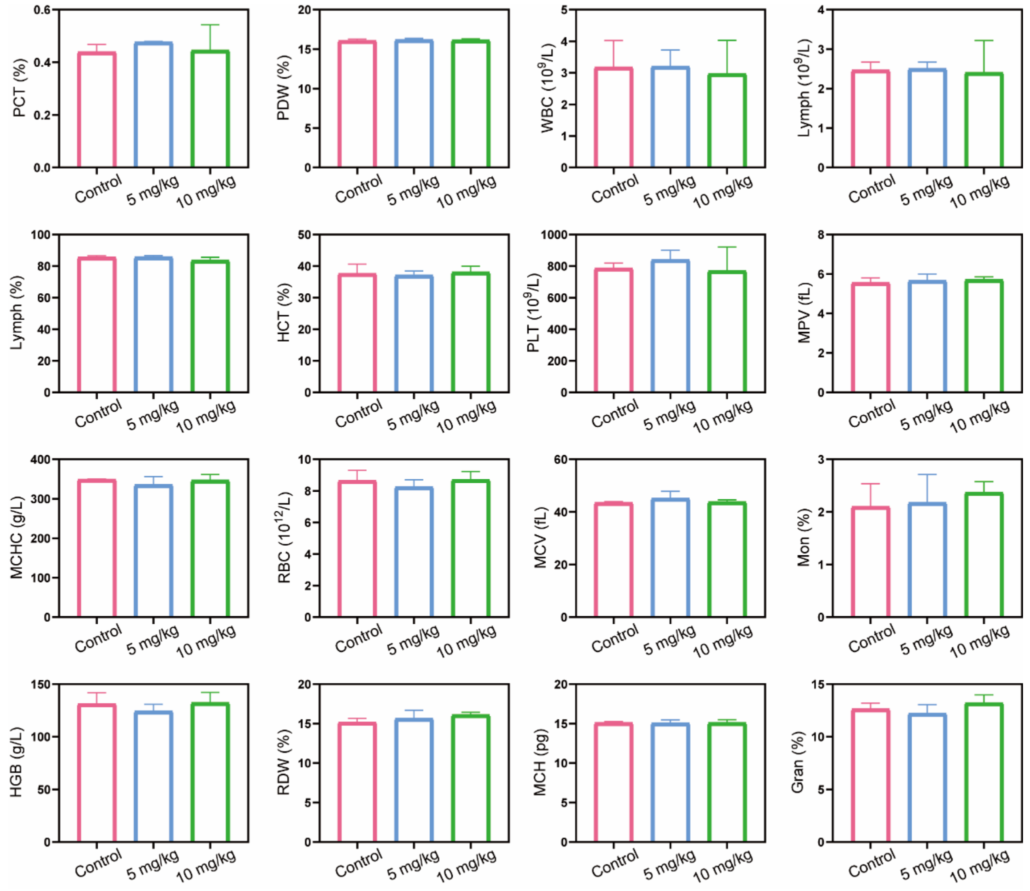

Figure S12. Routine blood indexes of the Kunming mice after intravenous injection of PBS (control) and Aza-BD@PC NPs at different concentrations, including platelet distribution width (PCT), platelet distribution width (PDW), white blood cell (WBC), lymphocytes (Lymph), lymphocyte ratio (Lymph%), hematocrit (HCT), platelet (PLT), mean platelet volume (MPV), mean corpuscular hemoglobin concentration (MCHC), red blood cell (RBC), mean corpuscular volume (MCV), monocytes (Mon), hemoglobin (HGB), red blood cell distribution width (RDW), mean corpuscular hemoglobin (MCH), and granulocytes (Gran) (n = 5). Data are represented as mean ± SD.

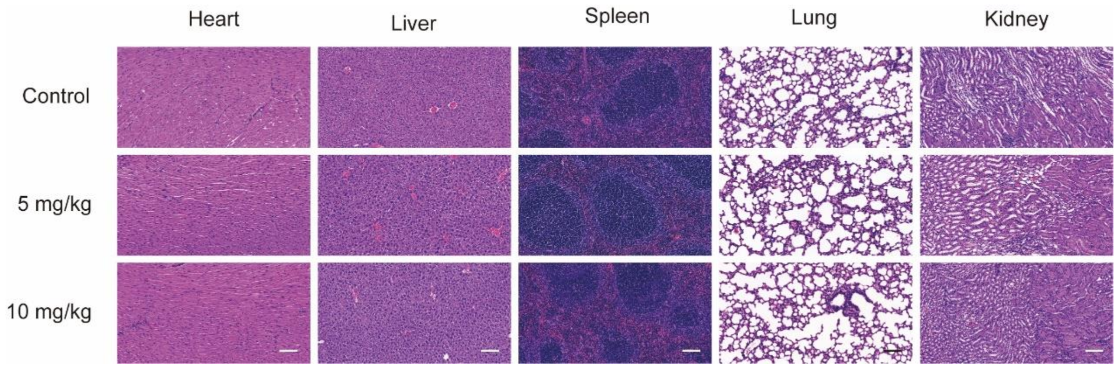

Figure S13. H&E staining images of the representative major organs from the Kunming mice after intravenous injection of PBS and Aza-BD@PC NPs at different concentrations for 30 days (scale bar: 200 μm).

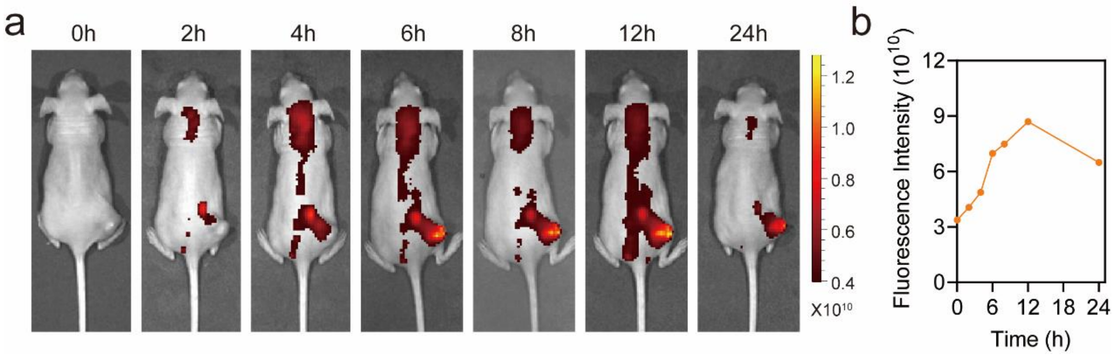

Figure S14. a) *In vivo* fluorescence images, and b) the quantitative analysis of the fluorescence intensity after intravenous injection of Aza-BD@PC NPs for various durations (n = 3). Data are represented as mean ± SD.

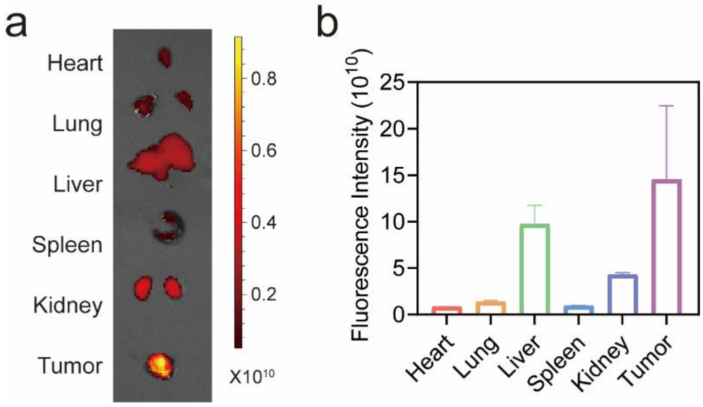

Figure S15. a) *Ex vivo* fluorescence images, and b) the quantitative analysis of the main organs and tumor tissues of the GL261 tumor-bearing mice at the end of the observation period (n = 3). Data are represented as mean ± SD.

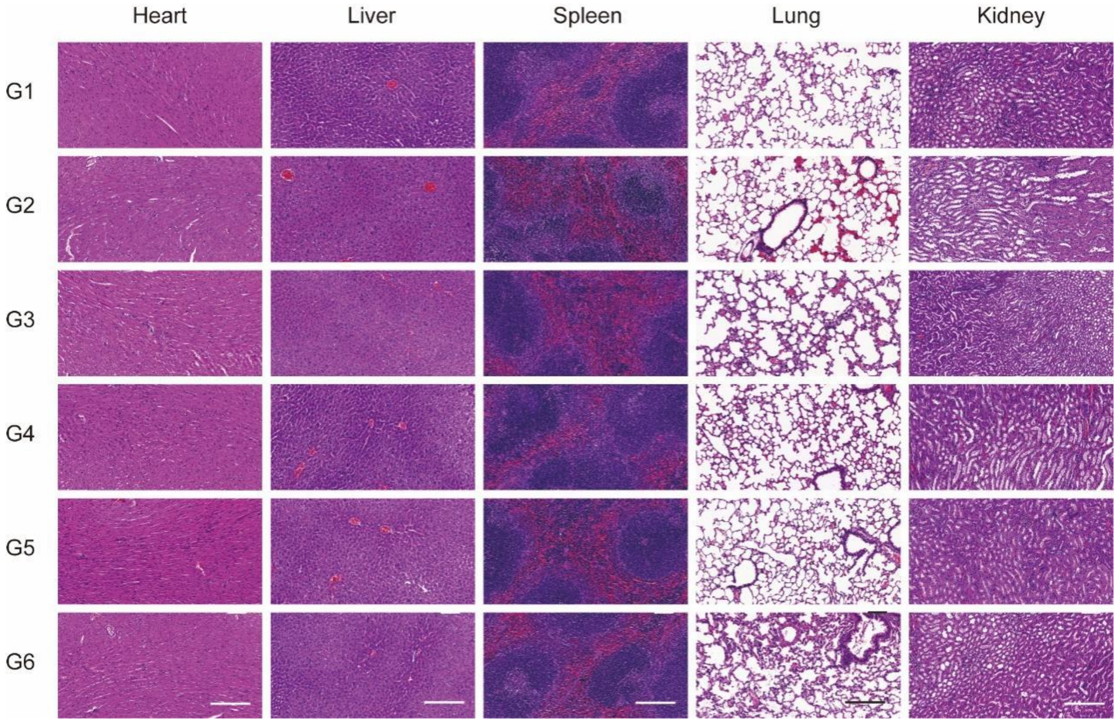

Figure S16. H&E staining images of the major organs from the GL261 tumor-bearing mice in various treatment groups (scale bar: 200 μm). G1: Control, G2: US, G3: Aza-BD NPs G4: Aza-BD NPs + US, G5: Aza-BD@PC NPs, G6: Aza-BD@PC NPs + US.

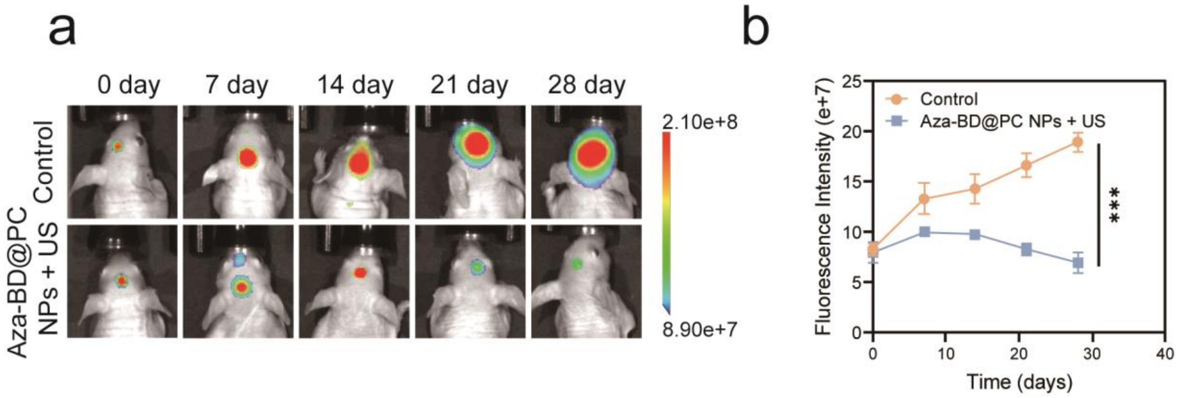

Figure S17. a) Intracranial tumor fluorescence images, b) quantitative analysis of fluorescence intensity (n = 3). Data are represented as mean ± SD and analyzed by one-way ANOVA. ns: no statistical difference, *$p < 0.05$, **$p < 0.01$, and ***$p < 0.001$.
